# Learning to live with ticks? The role of exposure and risk perceptions in protective behaviour against tick-borne diseases

**DOI:** 10.1371/journal.pone.0198286

**Published:** 2018-06-20

**Authors:** Daniel Slunge, Anders Boman

**Affiliations:** 1 Department of Economics, University of Gothenburg, Gothenburg, Sweden; 2 Gothenburg Centre for Sustainable Development, University of Gothenburg, Gothenburg, Sweden; University of Kentucky College of Medicine, UNITED STATES

## Abstract

The purpose of this study is to analyse the role of risk perceptions and exposure for protective behaviour against tick bites and the related diseases Lyme borreliosis (LB) and tick-borne encephalitis (TBE), both of which are growing health concerns. We use data from a national survey in Sweden with respondents in geographical areas with substantial differences in both abundance of ticks and incidence of LB and TBE. We find that the share of respondents who frequently use protective clothing (64%), perform tick checks (63%) or avoid tall grass while in areas with ticks (48%) is relatively high. However, the use of protective measures is uneven and a considerably lower share tuck their trousers into their socks (18%), use repellent against ticks (16%) or use a combination of protective measures. Thirty-one per cent of the respondents report one or more tick bites in the last year and 68% report one or more lifetime tick bites, indicating that it is difficult to protect oneself from tick bites. There is a strong positive association between exposure and checking the skin for ticks, but exposure is only weakly associated with other protective measures. Tick bites are perceived as a serious health risk by as many as 43% of the respondents. The perception that a single tick bite is serious is negatively associated with actual exposure to ticks, while the opposite is true for the perception that tick bites constitute a serious lifetime health risk. This indicates a learning effect in relation to risk perceptions and the performance of tick checks, but not in relation to other protective measures. Recommendations include informing people of the risks associated with tick bites, the efficacy of various protective measures and the importance of combining multiple types of protection. Given the high exposure to tick bites, the growing incidence of TBE and LB, and the difficulties in preventing tick bites, other preventive measures should be further discussed, including vaccination programmes.

## 1. Introduction

While risk perceptions play an important role in protective behaviour against various health risks [1–3], perceived risk is often inconsistent with objective measures of risk [4]. This inconsistency is especially common for new health risks perceived as difficult to control [4, 5] and may lead to levels of protection that are not optimal from an individual or a societal perspective.

The purpose of this study is to analyse the role of exposure and risk perceptions for protective behaviour against tick-borne diseases, which have become a growing public health problem in Europe and elsewhere. Partly due to climate change, ticks have spread to areas where they were not present earlier [6, 7] and the pathogens carried by ticks represent a new health threat in these regions. The incidence of the two most common tick-borne diseases—tick-borne encephalitis (TBE) and Lyme borreliosis (LB)–has increased in many countries [8, 9].

TBE is caused by the TBE virus, a flavivirus transmitted to humans by ticks that can cause severe infection of the central nervous system. Around 40% of those infected by the European subtype of the virus suffer from serious long-term or permanent sequelae [8, 10]. LB infection is caused by spirochetes belonging to the *Borrelia burgdorferi* sensu lato complex. The infection may affect several organs and tissues of the human body. While symptoms can be mild or absent for some individuals, they can be severe for others, especially if not treated at an early stage [9]. There is no cure for TBE but the disease can be effectively prevented by vaccine [11, 12]. The situation is the opposite for LB, i.e. there is no vaccine available on the market but the infection can be treated with antibiotics.

Risk assessment is complicated by the heterogeneous distribution of the TBE virus and different *Borrelia* species. While the mean prevalence of TBE virus in ticks in northern Europe is estimated at 0.28% and the mean prevalence of *Borrelia burgdorferi* species in ticks in 24 European countries is estimated at 14%, the regional variation in prevalence can be considerable [13, 14]. Despite a higher mean prevalence (26%) of ticks collected in Sweden that carried *Borrelia* bacteria, only 2% of those who had been bitten by a tick were diagnosed with LB [15, 16]. This indicates that that even after a bite by a tick that carries *Borrelia*, the risk of developing LB is low in each individual case. Nevertheless, given the large number of tick bites and the spread of ticks to new regions, this may still be a cause for concern.

Should public policy address this growing health threat more actively? Normally, public costs for health interventions need to be motivated by the avoidance of externalities (such as the spread of contagious diseases) or the provision of public goods (such as a healthy society). Because tick-borne diseases cannot be transmitted from one person to another, there is no positive external effect from individual vaccination (no so-called herd immunization) or other types of protective behaviour. Yet, if the costs to society caused by tick-borne disease are large, in a country with a publicly financed health system, public policy measures may still be motivated. Policy measures could also be justified for reasons sometimes referred to as paternalistic, i.e. the more informed regulator would encourage people to protect themselves out of concern for their health if the people for some reason do not protect themselves in a way that is optimal from a societal perspective [17].

One such reason could be the difficulties involved in assessing events with small probabilities but a potentially large impact, such as the risk of contracting a tick-borne disease. For such events, laypeople tend to focus more on the perceived severity of the event if it does occur, while experts focus more on the probability [4, 5, 18]. There is some evidence that an expert-layman divide exists in risk perceptions related to LB [19]. Risks related to ticks may also be overestimated due to perceptions that they are difficult to control, or because ticks cause feelings of disgust and are often portrayed in alarmist media headlines [4, 20–22]. While it is common that ‘risk alarmists’–people with high risk perceptions—are vocal in the public debate (see e.g. [23] in relation to LB), there is often a larger and more silent group of ‘risk deniers’–people with very low risk perceptions despite the fact that real risks do exist [22].

The most common policy measure to reduce the risk of tick-borne diseases is for health authorities to undertake information campaigns and education interventions aimed at increasing the use of various protective measures that individuals can undertake. Individual protective measures commonly recommended include avoiding risk areas or staying on trails while in risk areas, using protective clothing (long sleeves and trousers), tucking trousers into socks, using tick repellent, and checking the body for ticks and removing them before or as soon as possible after they attach [24, 25]. In countries where TBE is endemic, health authorities also commonly recommend vaccination against TBE for people in risk areas [11]. There is mixed evidence on the effectiveness of these protective measures. Protective clothing makes it more difficult for ticks to attach [25, 26], some repellents have been proved to deter ticks [27] and the risk of LB is reduced if attached ticks are removed within 24–48 hrs [27, 28]. However, only few studies using control trials on the effectiveness of protective clothing and tick checks in preventing tick bites exist. One such study finds evidence that protective clothing but not tick checks is effective in preventing tick bites [29]. Several studies find that vaccination is effective in preventing TBE [11, 30].

Despite the existence of risk-reducing measures, their use is uneven and can be surprisingly low in areas where ticks and LB are endemic [31–34]. Temporary visitors to endemic areas are more likely than full-time residents to undertake protective measures [31, 35]. A number of studies find only weak or ambiguous associations between exposure and protection [34, 36, 37]. This is surprising since the benefits of protection should increase with exposure to risk.

One possible explanation to the weak association between exposure and protection is that risk perceptions are dulled in endemic areas as people get used to living with the risk of tick-borne diseases and perceive them as less serious than residents in lower incidence areas [34] or temporary visitors [31, 35]. Several studies have found that the perceived risk of tick bites and LB have a stronger association with protective behaviour than does actual exposure to risk [34, 36, 37]. However, explaining protective behaviour with risk perceptions is complicated by a potential endogeneity problem [38]. While higher risk perceptions may lead to a higher use of protective measures, there may be important feedback mechanisms from this behaviour to risk perceptions. We discuss this further below.

A second explanation may be that the cost of using a protective measure is perceived to be greater than the benefit. Perceived costs of using protective measures against tick-borne diseases include discomfort (wearing protective clothing in summer is too warm), image issues (looking stupid with trousers tucked into socks), informational costs (not knowing how to remove a tick) and health risks from the use of repellents [21, 37]. Negative associations between the cost of using a protective measure and its use have been found in relation to several other health risks [39–41].

From a public health perspective, it is hence important to further understand how exposure and risk perceptions are associated with protective behaviour against tick bites and tick-borne diseases. Is increased exposure to risk associated with more frequent use of protective measures? Or is exposure associated with a downward adjustment in risk perceptions leading to an ambiguous association between exposure and protective behaviour? If the latter is true, risk perception is a poor predictor of protective behaviour in groups with high exposure.

In this paper, we try to answer these questions through a careful investigation of the associations between exposure, risk perceptions and protective behaviour within a large sample of respondents in Sweden. Sweden provides an interesting case study because of its large geographic variation in the abundance of ticks and the incidence of LB and TBE [6, 42].

Our analysis contributes to the existing literature in several ways. First, the exogenous geographic variation in the risk of contracting LB or TBE in the various areas of residence of our survey respondents enables us to analyse exposure, risk perceptions and protective behaviour in a variety of risk contexts. In this way, we partly address the potential endogeneity involved in explaining protective behaviour in connection with risk perceptions or exposure [38]. A similar approach was taken by [36], who compared risk perceptions and protective behaviour between respondents in an LB-endemic region in Switzerland and respondents in an emerging risk area in Canada. However, in our study, all respondents are in the same political and institutional context, reducing the potential confounding factors that can be found in cross-country studies.

Second, by using two distinct measures of risk perception, we show that, while the perceived seriousness of a single tick bite decreases with exposure and experience, the perceived lifetime health risk from tick bites increases with experience. Third, we contribute to the ambiguous literature on demographic factors associated with protective behaviour and identify groups of respondents who have high exposure but a low degree of protective behaviour. It may be particularly important to target such groups in risk management efforts by public authorities. Finally, despite the significant presence of ticks, LB and TBE in Sweden, surprisingly little is known about risk perceptions and protective behaviour. Stjernberg and Berglund (31) investigate protective behaviour on the small island of Aspö in southern Sweden, where LB and TBE are endemic. However, this is the first national survey and analysis of risk perceptions and protective behaviour related to ticks and tick-borne diseases in Sweden.

## 2. Data and methodology

### 2.1. Empirical strategy

Analysing the role of risk perception and exposure for these protective behaviours is complicated by a potential endogeneity problem [38]. While risk perception may be positively linked to protective behaviour, for example the use of protective clothing, there may be important feedback mechanisms from this behaviour to risk perceptions. This could also lead to risk compensation, where a perceived increase in the level of protection leads to increased exposure [43]. There may also be unobserved factors that affect protective behaviour against ticks, factors that may be correlated with risk perceptions, leading to omitted variable bias. Ignoring this potential endogeneity problem may lead to biased estimates of the effect of risk perception on protective behaviour. We partly address this problem by including exogenous variables in our analysis. These are demographic variables and variables capturing the level of risk of getting tick bites, LB and TBE when visiting tick habitats in various areas.

We focus on five different kinds of protective behaviour against ticks and tick-borne diseases: checking the skin for ticks after having spent time in tick habitats, using protective clothing (long sleeves and trousers), tucking trouser legs into socks, using insect repellent and avoiding tall grass and bushes while in areas with ticks. We also discuss associations between these behaviours and TBE vaccination.

Because earlier studies have shown that there are differences in the factors associated with distinct protective behaviours [36, 37], we first analyse each behaviour independently of the others. We use a logistic regression model with the following specification to analyse which explanatory variables are associated with each type of protective behaviour. In a first step, we analyse how a protective behaviour is associated with exposure and demographic variables:

$${protect}_{ij}=\beta_{0}+\beta_{1}D_{i}+\beta_{2}R_{i}+\beta_{3}E_{i}+u_{i}$$ (1)

where, in line with other recent studies [36, 37], *protect*_*ij*_ is a dummy variable equal to one if respondent *i* uses protective measure *j* often or always (and zero if never or rarely). *D*_*i*_ is a vector of demographic characteristics, *R*_*i*_ is a vector of objective risk variables in a geographical area and *E*_*i*_ is a vector of exposure variables. *u*_*i*_ is an error term.

Next, we expand the model by adding variables concerning risk perceptions (*P*_*i*_) and knowledge about ticks and tick-borne diseases (*K*_*i*_).

$${protect}_{ij}=\beta_{0}+\beta_{1}D_{i}+\beta_{2}R_{i}+\beta_{3}E_{i}+\beta_{4}P_{i}+\beta_{5}K_{i}+u_{i}$$ (2)

In a third step, we assess the robustness of our results by introducing a set of control variables. These include the perceived efficacy of protective measures, education, ownership of an outdoor pet, access to a summerhouse in a TBE risk area, TBE vaccination and work-related exposure to tick bites.

To analyse if the behaviours are implemented in combination or as substitutes, we also analyse the behaviours jointly using a count model as well as a multinomial logit model. First we use a dependent variable, *protect 0*–*5*, which is defined on a scale from zero (0) to five (5) depending on the number of protective measures used. To estimate the associations between this dependent variable and our independent variables, we use a Poisson count model. A limitation of the count model is that there is no ranking of the different measures, so that for example checking the body for ticks is ranked equally with using repellent or avoiding tall grass and bushes, even though checking the body for ticks may provide protection superior to the two other measures jointly. We compare the results from using *protect 0–5* as dependent variable with the results when using a somewhat different count variable, *protect 0–15*, as dependent variable. This variable also takes into account the frequency of the use of each protective measure (See S1 Table for variable definitions).

Second, we use a multinomial logit regression model to analyse associations with the most frequent combinations of protective measures. The dependent variable *protect MNL* is defined on a scale from zero (0) to nine (9) where 0 represents no protective measure used and values 1–8 represent those protective measures or combination of measures used most frequently. Value 9 indicates a combination of measures used infrequently (by less than 5% of the respondents) and is not analysed (See S1 Table).

**Fig 1:**
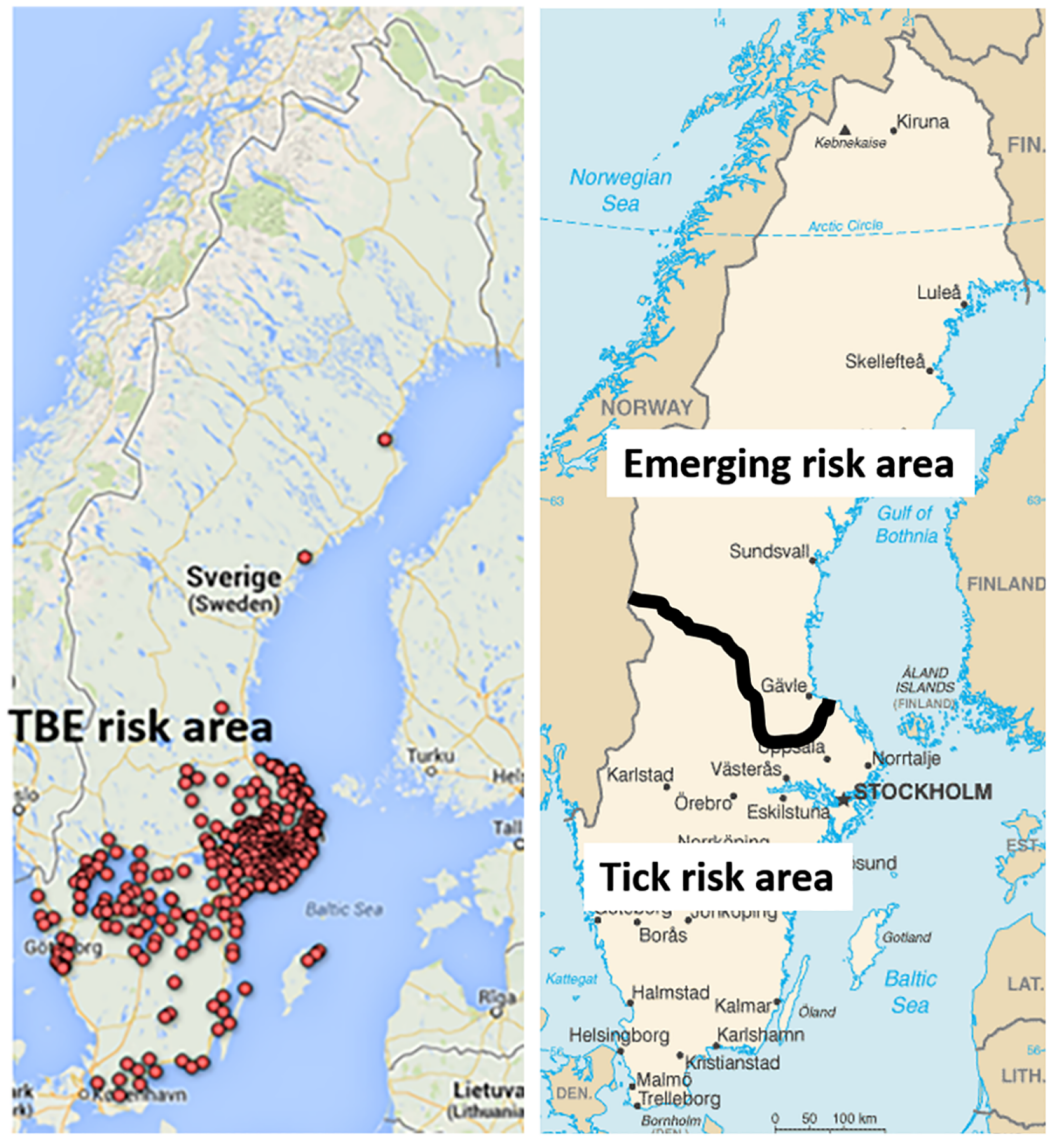
TBE risk area, tick risk area and emerging risk area. Each dot represents the geographical coordinates reported to the Swedish Public Health Agency for each of the 2 687 TBE cases in Sweden 1986–2012. Tick risk areas are in this study defined as areas situated south of Norrland that are not classified as TBE risk areas. The map to the left has been produced with google fusion tables.

**Fig 2:**
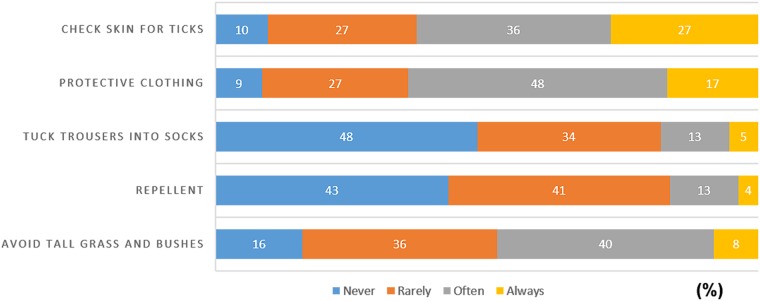
Share of respondents using different protective measures against ticks (n = 1510).

To account for the considerable heterogeneity in the risk of encountering ticks and getting infected with LB or TBE in Sweden, we classify the risk in the area of residence of the respondents into three categories. Fig 1 shows the geographical locations of these areas. Our identification of TBE risk areas is based on geographical data for the 2 687 TBE cases in Sweden 1986–2012 reported by the Swedish Public Health Agency, which we cluster in areas based on three-digit postal codes. We define *TBE risk areas* as areas with two or more reported cases of TBE in a three-digit postal code area in the years 1986–2012. This is similar to the classification of risk areas used by Swedish regional health authorities when producing TBE risk maps [44]. We define the *emerging risk area* as the geographical area of Norrland. In this area, which is situated north of the biogeographical boundary Limes Norrlandicus, there were no ticks in the past, but ticks have spread to the area in recent decades, partly as a result of an increasingly warmer climate [6, 45]. Remaining areas are defined as *tick risk areas*, that is areas situated south of Norrland that are not classified as TBE risk areas. Although this is a very rough division, it reflects the considerably longer history and higher presence of ticks and LB risk in tick risk areas than in the emerging risk area [6]. This classification of risk areas corresponds to the pattern of tick bites and experience with tick-borne diseases found in our data (see Fig 2 and Table 1).

**Table 1:** Descriptive statistics (mean values).

	(1)	(2)	(3)	(4)	(5)	(6)
VARIABLES	All respondents	Emerging risk area	Tick risk area	TBE risk area	Not TBE vaccinated	TBE vaccinated
Female respondent	0.54	0.55	0.54	0.53	0.54	0.54
Age^a^	50.9	50.3	50.5	51.9	50.1	54.2
Household pre-tax income/month (SEK)^b^	44.0	41.7	43.9	45.1	42.3	49.7
Has studied at university	0.52	0.50	0.55	0.47	0.60	0.50
Has child under 18 years	0.26	0.28	0.25	0.28	0.27	0.24
Lives in the countryside/small village	0.30	0.37	0.28^Ω^	0.31	0.33	0.24
Monthly or more frequent visits to areas with ticks	0.83	0.70^#^	0.84	0.87	0.80	0.93
Monthly or more frequent visits to areas with risk of TBE	0.37	0.13*	0.35*	0.53*	0.28	0.67
Has had 1 or more tick bites in lifetime	0.68	0.25^#^	0.73	0.76	0.64	0.85
Had at least 1 tick bite in last 12 months	0.31	0.04^#^	0.34	0.37	0.26	0.48
Respondent has had a tick-borne disease	0.12	0.02^#^	0.13	0.14	0.09	0.20
Family member/close friend has had a tick-borne disease	0.41	0.18^#^	0.43	0.46	0.36	0.56
Perception: tick bites rather or very high risk to health	0.43	0.19*	0.44*	0.50*	0.38	0.60
Perception: rather or very serious to get tick bite	0.42	0.42	0.42	0.42	0.41	0.43
No. of correct answers on knowledge questions (0–7)	3.82	2.87^#^	3.92	4.03	3.58	4.73
No. of protective measures used often/always (0–5)	2.10	1.74^#^	2.10	2.24	2.06	2.26
Check body for ticks	0.63	0.29^#^	0.67	0.70	0.59	0.77
Covering clothing	0.64	0.64	0.64	0.66	0.65	0.64
Tuck trousers into socks	0.18	0.18	0.16	0.21	0.19	0.18
Repellent	0.16	0.21	0.15^Ω^	0.16	0.15	0.19
Avoid tall grass and bushes while in areas with ticks	0.48	0.42	0.48	0.50	0.48	0.49
Vaccinated against TBE	0.24	0.07*	0.23*	0.35*	0	1
Observations^c^	1510	187	884	439	1113	361

Notes:

^a^ Age: the standard deviation among all respondents is 17.0 years; min = 18 years; max = 80 years;

^b^ Income: the standard deviation among all respondents is SEK 23 000; min = SEK 5 000; max = SEK 115 000. Respondents indicated their income in intervals of SEK 10 000. The average income is generated from the mean in each interval.

^c^ The number of observations was 1 502 for *has studied at university*, 1 507 for *family member/close friend has had a tick-borne disease* and 1 474 for *vaccinated against TBE*.

*Mean estimates for the different risk areas are significantly different from each other (p<0.05, Pearson Chi-square statistic).

^#^ Mean estimate is significantly different from other risk areas (p<0.05, Pearson Chi-square statistic).

^Ω^ Mean estimate is significantly different from emerging risk area (p<0.05, Pearson Chi-square statistic).

### 2.2. Data collection

A questionnaire (S1 Text) was developed based on focus group discussions, two pilot tests and key informant interviews with doctors and epidemiologists specialising in tick-borne diseases. The survey was performed under informed consent and approved by the Regional Ethical Review Board at the University of Gothenburg (decision number 544–13). The design of the questionnaire was informed by earlier studies on variables associated with protective behaviour and risk perceptions and included questions about experience, exposure, risk perception, knowledge and protective behaviour related to ticks and tick-borne diseases, as well as socio-economic information about the respondent and his/her household.

The questionnaire was distributed online in October 2013 to 6 000 respondents aged 18–85 years in a national internet panel representative of the Swedish population. The internet panel consists of approximately 8 000 members recruited through telephone interviews with randomly sampled respondents (selection into the sample is therefore reduced compared with e.g. a voluntary opt-in survey). After two reminders, responses from 2 066 participants were received, corresponding to a response rate of 34%. This paper uses only the 1 510 respondents (25%) who answered all questions corresponding to the variables included in this analysis.

The low response rate raises concerns about potential sample selection bias. Web-panel respondents may, for example, spend less time outdoors than the population in general and would hence be less exposed to the risk of tick bites. Another possibility is that the respondents are more concerned about ticks and related diseases and more likely to exhibit protective behaviour than the population average. Because this is the first national study in Sweden on protective behaviour against ticks and tick-borne diseases, there are no good comparative statistics for many of our variables. However, we can compare the share of vaccinated respondents in our study with a recent study of TBE vaccination rates in Stockholm County [46], which finds that 53% of the population in Stockholm County has ever had a TBE vaccine shot. TBE is endemic to Stockholm County, and it is expected that the vaccination rate within its borders is considerably higher than the Swedish average. In our study, 24% of all respondents and 48% of the respondents living in Stockholm County were vaccinated. This indicates that our study found approximately the same vaccination rate as the survey used by Askling, et al. (46). The large share of the respondents in the survey who engage in outdoor activities very frequently also corresponds to findings about outdoor habits from other surveys of the Swedish population [47]. This reduces our concerns about the response rate.

## 3. Results

### 3.1. Descriptive statistics

Definitions and summary statistics of the independent variables used in the analysis are provided in Table 1 and S1 and S2 Tables. We use the variance inflation factor (VIF) to control for potential multicollinearity between the independent variables. Mean VIF when all control variables are included in the regression analysis is 2.8. The highest VIF value is 7.1, which is found for the knowledge variable. This is below 10, which is the standard benchmark for multicollinearity.

Column 1 reports summary statistics for all respondents. In Columns 2–4, respondents are divided into three groups according to the prevalence of ticks, LB and TBE in their area of residence. Of the 1 510 respondents in the sample, 12% live in the emerging risk area, 59% live in tick risk areas and 29% live in TBE risk areas. Columns 5–6 report summary statistics for respondents vaccinated/not vaccinated against TBE, respectively.

We find some small but statistically significant differences in socio-economic characteristics between our survey respondents and the Swedish population. Using a t-test, we cannot reject the hypothesis of equal mean values between the sample and the population. In 2013, the mean age in the population was 49 and 51 in the sample. The share of women was 50% in the population and 54% in the sample. The mean monthly household income was SEK 40 600 in the population and SEK 44 000 in the sample (Statistics Sweden, 2013). Based on a comparison with geographically coded population statistics, we find that the geographical distribution of the respondents is largely representative of the Swedish population. In Section 4, we discuss possible implications of these differences for our results.

A large share of the respondents state that they often or always check their body for ticks after being outdoors in areas with ticks (63%), use protective clothing (64%) or avoid tall grass or bushes when in forests or other areas with ticks (48%). A much lower share often or always tuck their trousers into their socks (18%) or use repellent (16%) as protective measures. There is also a large share of the respondents stating that they never use any of these two protective measures (see Fig 2).

Considerably fewer respondents use a combination of several protective measures. Forty-five per cent of the respondents use protective clothing *and* perform tick checks, and 15% use these two measures in combination with tucking their trousers into socks. Four per cent report that they use all five of these protective measures often or always. Twenty-four per cent were vaccinated against TBE.

The use of tick checks and protective clothing found in this study is somewhat higher and the use of repellent lower than in the LB- and TBE-endemic Swiss region Neuchâtel [36]. It is also considerably higher than in the Netherlands, where, according to Beaujean, et al. (37), 37% use protective clothing and 32% check their bodies for ticks. One possible explanation for the considerably higher use of protective measures in Sweden than in the Netherlands is the higher exposure to ticks. Sixty-eight per cent of the respondents in our sample had been bitten by one or more ticks, compared with 21% in the study from the Netherlands.

Spending time outdoor in forests or other areas where there may be ticks is very common, with 83% of the respondents reporting spending time in such areas on a monthly or more frequent basis from May to September. Thirty-seven per cent of the respondents report spending time in areas where they know the ticks may be infected with TBE.

Experience with tick bites and tick-borne disease is common among the respondents. Only 32% reported they had never had a tick bite, 11% reported to have been diagnosed with LB and 1% had been diagnosed with TBE or other tick-borne diseases. Because there is no requirement in Sweden to notify public health authorities about LB cases, there are no comparative disease statistics. A study of a highly LB-endemic area in Sweden found that 25% of the respondents had been treated for LB at least once [31]. In the LB-endemic region of Neuchatel in Switzerland, Aenishaenslin, et al. (19) found that 6% had been diagnosed with LB. Forty-one per cent of the respondents report they have a family member or a close friend who has had a tick-borne disease.

The average perceived risk concerning ticks and tick-borne diseases is very high. Forty-two per cent perceive that it is rather or very serious to be bitten by a tick, and 43% of the respondents answered that tick bites generally constitute a rather large or very large risk to his/her health or the health of his/her family. In comparison, 26% and 31% answered that air pollution and traffic accidents, respectively, constitute a rather or very large risk.

**Fig 3:**
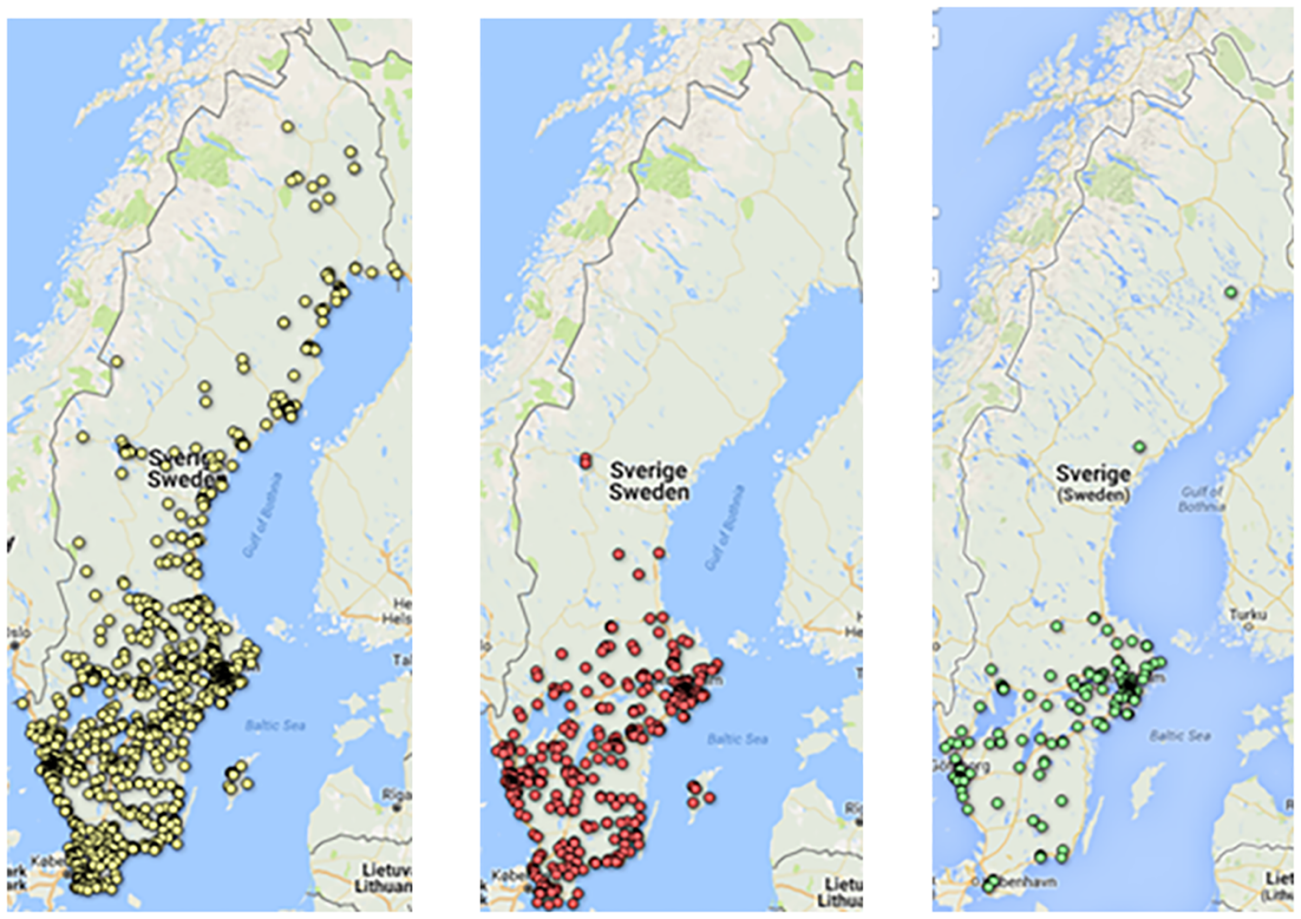
Geographical location of respondents’ place of residence, reported tick bites and TBE-vaccinated respondents. (A) Place of residence of respondents (n = 1510). (B) Place of tick bite in last 12 months reported by respondents (n = 615). (C) Place of residence of TBE-vaccinated respondents (n = 362). The maps have been produced by adding survey data to google maps using google fusion tables.

Comparing respondents in the different risk areas (Table 1, Columns 2–4), we find notable differences regarding exposure, risk perceptions and knowledge. As expected, tick bites are mainly experienced in tick-risk areas and TBE risk areas, with only 4% of respondents in the emerging risk area reporting at least one tick bite in the last 12 months. In comparison, 34% of the respondents living in tick risk areas and 37% living in TBE risk areas reported one or more tick bites in the last 12 months. In addition, experience with and knowledge about tick-borne diseases increase with the level of risk in the area of residence. Fig 3 illustrates the geographical location of the area of residence of the respondents, places where respondents report they were bitten by ticks in the previous year and the area of residence of TBE-vaccinated respondents.

Considering the large difference in experience with ticks, there is surprisingly little difference between the shares of the respondents in the different risk areas who use protective measures. Besides TBE vaccination, checking the body for ticks after being outdoors is the only protective measure used significantly more in tick and TBE risk areas than in the emerging risk area. We find no significant differences between respondents in the emerging risk area and in the other risk areas in their use of protective clothing, tucking trousers into socks or avoiding tall grass and bushes. The use of repellent is significantly higher in the emerging risk area than in tick risk areas, indicating that respondents use—or have become accustomed to use—repellent for other reasons than ticks, for example as protection against mosquitos. This could also be true for other protective measures. In a study of protective measures in the UK, frequent use of long trousers was primarily due to factors such as the weather or avoidance of cuts and scrapes and not to an intention to prevent tick bites [21]. The only statistically significant difference between respondents in tick risk areas and TBE risk areas is found in relation to TBE vaccination and tucking trousers into socks.

Statistically significant differences between TBE vaccinated and unvaccinated respondents are discussed in Slunge (48). Regarding protective behaviour, we find that checking the body for ticks is a significantly more frequent behaviour among vaccinated respondents (p<0.01, Pearson Chi-square statistic). Also the use of repellent is more common among vaccinated respondents (p = 0.09, Pearson Chi-square statistic). We find no significant differences in relation to the other three protective behaviours (Table 1, columns 5–6).

### 3.2. Exposure, risk perceptions and protective behaviour

Table 2 reports results on variables associated with the five forms of protective behaviour. In columns 1–5, each of the five protective measures is estimated separately with logit. In column 6, the count variable *protect 0–5* is estimated with a Poisson count model. Following Eq 2, explanatory variables include demographic characteristics, exposure, risk perceptions and knowledge. In S3 and S4 Tables, results are reported with only demographic and exposure variables as explanatory variables (Eq 1) as well as with control variables included.

**Table 2:** Analysis of protective behaviour; marginal probabilities evaluated at sample means.

	(1)	(2)	(3)	(4)	(5)	(6)
VARIABLES^c^	Check skin^a^	Prot. Clothing^a^	Socks^a^	Repellent^a^	Avoid^a^	Protect 0–5^b^
Female respondent	0.128***	0.041	0.186***	0.101***	0.058**	0.488***
	(0.029)	(0.027)	(0.019)	(0.020)	(0.028)	(0.068)
Age 18–30	-0.031	-0.097**	-0.032	-0.064***	-0.032	-0.238**
	(0.048)	(0.047)	(0.027)	(0.024)	(0.048)	(0.103)
Age 46–65	-0.035	-0.024	-0.005	-0.080***	-0.059	-0.203**
	(0.041)	(0.038)	(0.026)	(0.022)	(0.040)	(0.088)
Age > 65	-0.092**	-0.032	-0.043	-0.079***	-0.064	-0.295***
	(0.045)	(0.041)	(0.026)	(0.024)	(0.043)	(0.093)
Household pre-tax income/ month (SEK 1 000)	-0.001	-0.002***	-0.001**	-0.000	-0.001	-0.004***
	(0.001)	(0.001)	(0.000)	(0.000)	(0.001)	(0.001)
Has child under 18 years	0.015	-0.025	-0.004	-0.046**	0.007	-0.067
	(0.037)	(0.035)	(0.024)	(0.023)	(0.038)	(0.085)
Lives in the countryside/small village	-0.036	-0.021	0.004	-0.022	-0.124***	-0.167**
	(0.031)	(0.028)	(0.019)	(0.019)	(0.029)	(0.068)
Monthly or more frequent visits to areas with ticks	0.122***	0.082**	-0.009	0.023	-0.059	0.150
	(0.042)	(0.038)	(0.027)	(0.024)	(0.039)	(0.102)
Monthly or more frequent visits to areas with risk of TBE	0.088***	-0.066**	0.008	0.024	-0.028	0.018
	(0.030)	(0.028)	(0.020)	(0.021)	(0.030)	(0.068)
1 tick bite in lifetime	0.082**	0.031	0.026	-0.006	0.045	0.184
	(0.039)	(0.042)	(0.033)	(0.030)	(0.046)	(0.121)
2–10 tick bites in lifetime	0.199***	-0.003	0.035	-0.008	0.020	0.235***
	(0.031)	(0.034)	(0.025)	(0.024)	(0.036)	(0.091)
>10 tick bites in lifetime	0.290***	-0.040	0.066*	-0.002	-0.096**	0.237**
	(0.028)	(0.044)	(0.037)	(0.030)	(0.045)	(0.117)
Lives in tick risk area	0.184***	-0.019	-0.047	-0.082***	0.058	0.102
	(0.046)	(0.042)	(0.031)	(0.031)	(0.045)	(0.122)
Lives in TBE risk area	0.168***	0.013	-0.005	-0.067**	0.089*	0.208
	(0.043)	(0.046)	(0.031)	(0.027)	(0.050)	(0.137)
Perception: tick bites rather or very high risk to health	0.132***	0.056**	0.016	-0.012	0.054*	0.207***
	(0.029)	(0.028)	(0.019)	(0.020)	(0.030)	(0.069)
Perception: rather or very serious to get tick bite	0.102***	0.078***	0.058***	0.027	0.168***	0.397***
	(0.029)	(0.027)	(0.020)	(0.020)	(0.028)	(0.070)
No. of correct answers on knowledge questions	0.033***	0.016**	0.006	0.017***	0.011	0.077***
	(0.008)	(0.008)	(0.005)	(0.006)	(0.008)	(0.020)
Observations	1510	1510	1510	1510	1510	1510
Pseudo-R2	0.19	0.03	0.11	0.05	0.05	0.034

Notes: Robust standard errors in parentheses.

*** p<0.01.

** p<0.05.

* p<0.1.

^a^ Dummy variable, estimated with logit.

^b^ Count variable 0–5 estimated with Poisson. A goodness-of-fit chi-squared test is not statistically significant indicating that a Poisson model fits the data. We also control for over dispersion by running the same regression model using negative binomial distribution.

^c^ See S3 and S4 Tables for models with only demographic and exposure variables as well as with control variables.

We find statistically significant and positive associations between all the exposure variables in the model—visits to areas with ticks and/or TBE risk, residing in tick risk or TBE risk area and experience with tick bites—and checking the body for ticks. The strength of the associations increases with the number of tick bites experienced.

We do not find similar strong positive associations between exposure and the other protective measures: While monthly or more frequent visits to areas with ticks is positively associated with the use of protective clothing, there is a negative association between visits to areas with TBE risk and the use of protective clothing. Having had more than 10 lifetime tick bites is the only exposure variable that is significantly associated with tucking trousers into socks (at the 10 per cent level). The use of repellent is negatively associated with residing in tick risk areas or TBE risk areas. Living in a TBE risk area is weakly positively associated with avoiding tall grass or bushes. Having had more than 10 tick bites and living in a rural area is negatively associated with avoiding high grass or bushes while in areas with ticks.

We find significant positive associations between exposure to tick bites and the count variable *protect 0–5* (column 6). This reflects the positive association between exposure and checking the body for ticks.

Turning to knowledge and risk perceptions, we find that a higher score on the seven knowledge questions is positively associated with tick checks, protective clothing and the use of repellent but not with the other protective measures. A perception that tick bites constitute a rather large or very large risk to the health of the respondent or the respondent’s family is positively and significantly associated with tick checks, using protective clothing and avoiding tall grass and bushes. There is also a positive and statistically significant association between perceiving that it is rather or very serious to get a tick bite and the use of all of the protective measures except for repellent. We find significant positive associations between knowledge and the count variable *protect 0–5* as well as between the two risk perception variables and *protect 0–5* (column 6).

**Fig 4:**
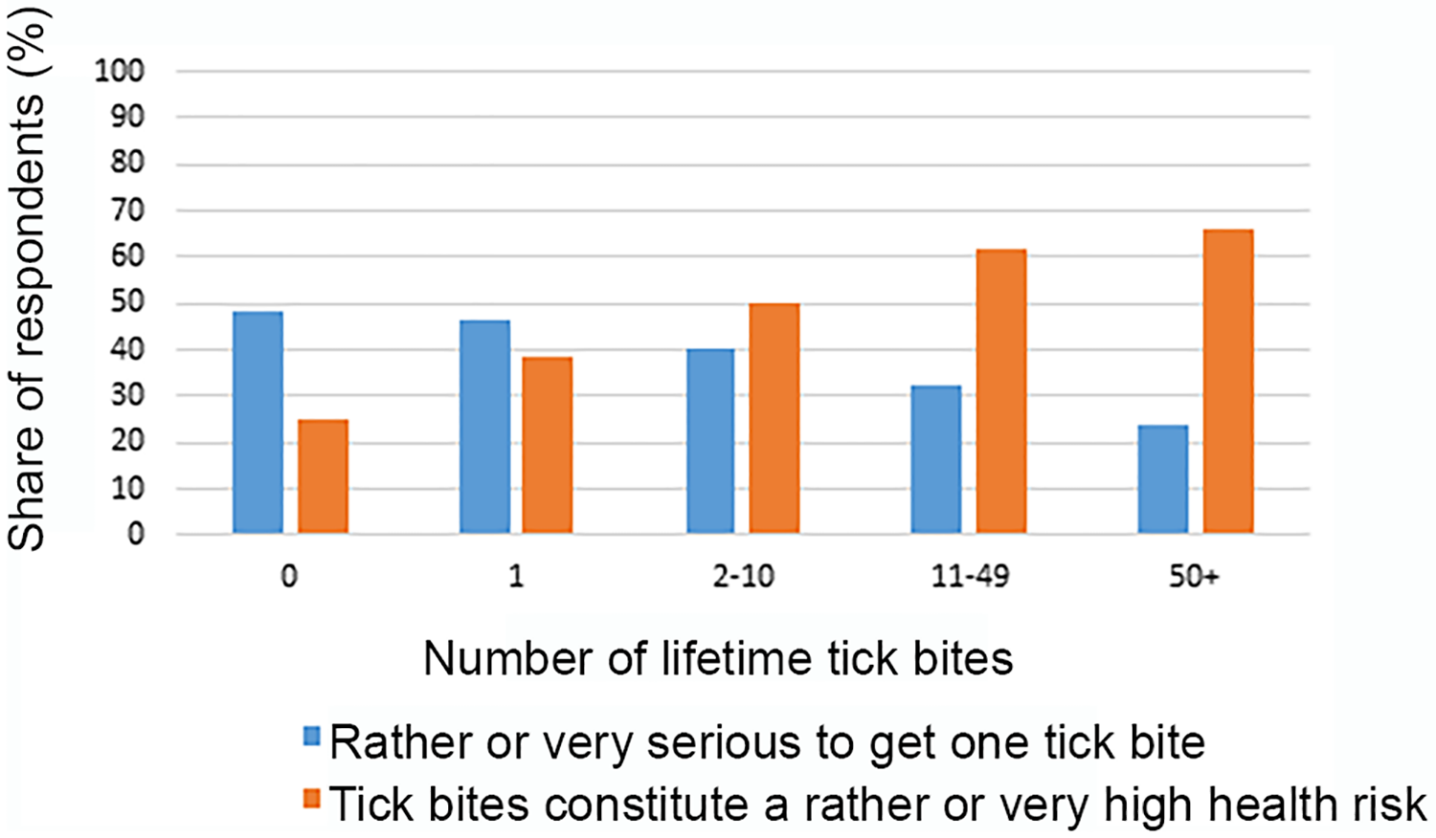
Risk perception and experience with tick bites.

However, there are important differences between how our different definitions of risk perceptions are associated with protective behaviours and exposure to ticks. While there is a significant *negative* association between exposure to ticks and the perceived seriousness of a *single* tick bite, there is a significant *positive* association between exposure and the perceived *lifetime* health risk from tick bites (Fig 4 and S5 Table). This indicates that people get used to having tick bites and adjust their risk preferences accordingly. They seem to learn that the probability of falling ill from a single tick bite is low, yet perceive that the cumulative effect of repeated tick bites constitutes a serious health risk. Frequent visits to areas with TBE risk is significantly and positively associated with both of the two risk perception variables, indicating that respondents perceive that a bite from a tick is more serious if received in an area with TBE risk (S5 Table).

We assess the robustness of our findings in several ways. In S3 Table, we find out whether the results reported above remain valid when a set of control variables are included in the model. While parameter estimates for the different measures of exposure and risk perception change when control variables are included, these changes are moderate and with few exceptions the significant associations reported above remain valid.

Among the control variables, we find expected positive associations between a perception that a specific protective measure is very effective and its use. This confirms findings from earlier studies [36, 37]. We also find a negative association between having a cat, dog or other outdoor pet and the use of protective clothing, tucking trousers into socks and repellent. Among the control variables, we also find a positive association between having a job where there is a risk of getting bitten by ticks and the use of protective clothing. We find no significant association between being vaccinated against TBE and other protective behaviours in the multivariate analysis.

In S4 Table, we compare the results for the model with the count variable *protect 0–5* as dependent variable with those for the model with the count variable *protect 0–15* as dependent variable. The latter variable also includes the frequency of usage of each protective measure. The two count variables produce similar results.

In S6 Table, we use a multinomial logit model to estimate the use of protective measures separately and in combination. This specification confirms the results reported above. We find significant positive associations between exposure and the use of tick checks only or tick checks in combination with protective clothes and/or socks and repellent. There is also a positive association between frequent visits to areas with TBE risk and a combination of checking the body for ticks and avoiding tall grass and bushes while in these areas. Exposure is significantly negatively associated with the use of protective clothing as the only protective measure as well as with a combination of protective clothes and avoidance of tall grass and bushes when visiting areas with ticks. Combinations of protective measures not involving tick checks are infrequent.

We also control if our results are sensitive to how we have defined the dummy variables for the protective measures. In S7 Table, we define our dummy variables for protective measures as 1 only if the protective measure is used always and as 0 otherwise. We find similar associations between the use of protective measures and our explanatory variables as reported in Table 2 using this alternative definition.

### 3.3. Demographic factors and protective behaviour

We find significant associations between gender, age, income and protective behaviour (Table 2). On average, women consistently use protective measures to a greater degree than men, perceive a higher level of risk and are more knowledgeable about tick-borne diseases. These differences are significant at the 1 per cent level in a univariate analysis (Pearson chi test, p<0.01). We find no significant differences between men and women regarding exposure. Several other studies also find that men are less likely than women to check their skin for ticks [31, 33, 36], but other studies find no such associations [21, 37].

We find that respondents older than 65 years are less likely than younger ones to conduct tick checks and that 18–30-year-old respondents are less likely than older age groups to use protective clothing (the reference group is *age 31–45*). This may be caused by increased costs due to taste preferences regarding appearance among young age groups and increased effort due to difficulties performing full body tick checks among the elderly. Respondents in the 31–45 age group are more likely to use repellent than other age groups. Aenishaenslin, et al. [36] also find a negative association between youth and the use of protective clothing, but most other studies do not find associations between age and protective behaviour. Our finding that income is negatively associated with the use of protective clothing corresponds to the finding that being unemployed is positively associated with the use of protective clothing [37]. Higher income is also known to be associated with lower risk perceptions [22]. Having a child below age 18 in the household is negatively associated with frequent use of tick repellent. Respondents living in the countryside are less likely to avoid tall grass and bushes when in areas with ticks. Using a multinomial logit model, we also find a positive association between living in a rural area and using protective clothing as the only protective measure (S6 Table).

### 3.4. Protective behaviour of highly exposed persons

From a risk management perspective, it is important to analyse the behaviour of groups that are particularly exposed to risk. We find that 12% of the respondents never or rarely check their body for ticks despite visiting areas with ticks weekly or daily and having experienced one or several tick bites. Similarly, 18% of the respondents never or rarely use protective clothing despite this high exposure. Six per cent of the high exposure respondents neither use protective clothing nor check ticks often or always. Four per cent never or rarely use any protective measure.

Table 3 reports factors associated with low use of skin checks and protective clothing for the group of high-exposure respondents. Columns 1 and 3 include demographic variables and variables related to the risk in the area of residence. In columns 2 and 4 we add explanatory variables related to risk perceptions, knowledge, the perceived efficacy of protective measures and TBE vaccination.

**Table 3:** Factors associated with high exposure^a^ and low protection^b^. (marginal probabilities after logit evaluated at sample means).

	(1)	(2)	(3)	(4)
VARIABLES	Check skin never/rarely	Check skin never/rarely	Prot.Clothes never/rarely	Prot.Clothes never/rarely
Female respondent	-0.055***	-0.037**	-0.044**	-0.044**
	(0.017)	(0.016)	(0.020)	(0.020)
Age	0.000	0.000	-0.000	0.000
	(0.001)	(0.001)	(0.001)	(0.001)
Household pre-tax income/month (SEK)	0.001**	0.001	0.001***	0.001
	(0.000)	(0.000)	(0.000)	(0.000)
Has child under 18 years	-0.018	-0.012	-0.012	0.000
	(0.019)	(0.018)	(0.022)	(0.022)
Lives in the countryside/small village	0.052***	0.058***	0.020	0.023
	(0.020)	(0.020)	(0.022)	(0.022)
Cat owner	-0.008	-0.010	0.041	0.050*
	(0.021)	(0.020)	(0.029)	(0.030)
Dog owner	0.064**	0.070***	0.041	0.029
	(0.025)	(0.025)	(0.027)	(0.026)
Other outdoor animal	0.020	0.011	0.138**	0.143**
	(0.044)	(0.040)	(0.063)	(0.071)
Lives in tick risk area	0.014	0.026	0.157***	0.117***
	(0.025)	(0.025)	(0.037)	(0.038)
Lives in TBE risk area	0.032	0.045	0.204***	0.135**
	(0.030)	(0.032)	(0.058)	(0.056)
Perception: tick bites rather or very high risk to health		-0.024		0.042**
		(0.016)		(0.020)
Perception: rather or very serious to get tick bite		-0.032**		-0.038**
		(0.016)		(0.019)
No. of correct answers on knowledge questions		0.000		0.013**
		(0.004)		(0.006)
Perception: Checking body for ticks is very effective protection		-0.051***		-0.010
		(0.019)		(0.021)
Perception: Protective clothing is very effective protection		0.024		-0.079***
		(0.017)		(0.019)
Vaccinated against TBE		0.029		0.103***
		(0.021)		(0.026)
Protective clothing		-0.063***		
		(0.017)		
Check body for ticks				-0.025
				(0.021)
Observations	1510	1473	1510	1473
Pseudo-R2	0.034	0.067	0.038	0.085

Notes: Robust standard errors in parentheses.

*** p<0.01.

** p<0.05.

* p<0.1.

^**a**^ High exposure is defined as visiting forests or other areas with ticks weekly or daily during the period May–September and having had at least one lifetime tick bite.

^**b**^ Low protection is defined as never or rarely conducting tick checks (for the dependent variable in columns 1 and 2) and never or rarely using protective clothing (columns 3 and 4) when in areas with ticks.

In line with the results reported above, we find that men are more likely not to use these protective measures despite high exposure. Income is also positively associated with high exposure and low protection, but this association disappears when risk perception and TBE vaccination variables are included.

Having a dog as well as residing in a rural area is positively associated with high exposure and no or infrequent tick checks. Having a cat or an outdoor animal—this may be a horse or a farm animal—is positively associated with high exposure and no or infrequent use of protective clothing. Living in a tick risk area or TBE risk area is also positively associated with high exposure and no or infrequent use of protective clothing.

There is a significant negative association between a perception that it is serious to get a tick bite and belonging to the low protection/high exposure group. There is also a negative association between the perceived efficacy of using the protective measure and belonging to the low protection/high exposure group. Surprisingly, we find that the low use of protective clothing/high exposure group is positively associated with knowledge about ticks, as well as with the perceived health risk from multiple tick bites. In line with earlier results [32] this indicates that increased knowledge and a general awareness of tick-borne diseases is not enough to make high-exposure people use protective clothing.

We also find that TBE-vaccinated respondents are 10 percentage points more likely to belong to the group of high-exposure respondents who never or rarely use protective clothing. This indicates that there is a share of the population who see TBE vaccination as a substitute for using protective clothing. The negative association between the use of protective clothes and infrequent tick checks indicates that these protective behaviours are complements and not substitutes in groups with high exposure.

## 4. Discussion

In this paper, we have analysed the role of risk perception and exposure for protective behaviour against tick bites, Lyme borreliosis (LB) and tick-borne encephalitis (TBE). We use empirical data from a national survey in Sweden with respondents in geographical areas differing in abundance of ticks and incidence of LB and TBE.

Outdoor recreation in forests and other areas with ticks is very common in Sweden. Also using protective measures against tick bites is frequent with over 60% of the respondents using protective clothing or checking their skin for ticks ‘often’ or ‘always’ in relation to visits to forests or other areas with ticks. However, despite the widespread use of these protective measures, experience with tick bites is high among the respondents, including in the last year. This indicates that it is difficult to protect oneself from tick bites.

The low share of respondents who use repellent (16%) or tuck their trousers into their socks (18%) or who use a combination of protective measures may partly explain the many tick bites reported in this study. The difference between using a protective measure often or always may also explain some of the exposure to ticks reported. Only 17% of the respondents report that they always use protective clothing when in areas with ticks and 27% that they always perform tick checks. There is also a segment of respondents who, despite very high exposure, never or rarely check their skin for ticks (12% of the respondents) or use protective clothing (18%).

The use of protective measures is associated with demographic factors. We find that men on average are less likely than women to use protective measures against ticks. While this finding is in line with the general risk perception literature showing that women tend to perceive risks as more serious [4, 5], earlier literature on gender and protective behaviour against ticks is ambiguous. Our finding that people younger than 30 are less likely to use protective clothing and that people older than 65 are less likely to perform tick checks may also be important from a health communication perspective.

There is a strong positive association between different measures of exposure—visits to tick and TBE areas, residence in risk areas and experience with tick bites—and checking the skin for ticks. However, we find only weak associations between exposure and other protective measures. Earlier studies also find that experience with tick bites is a significant determinant of checking the skin but not a predictor of the use of protective clothing [37]. This suggests that there is a strong learning effect regarding tick checks but not regarding protective clothing and that checking the skin for ticks is a more easily adopted measure than other ways of preventing tick bites [49, 50]. The cost of protection may also partly explain these findings. Using protective clothing on a warm summer day may be perceived as a high cost compared with checking the skin for ticks. Younger age groups may perceive a high ‘image cost’ from using protective clothing and older people may find it difficult or costly to conduct tick checks.

The perceived risk concerning ticks and tick-borne diseases is very high among the respondents. Forty-two per cent of the respondents perceive that being bitten by a tick is rather or very serious. The share of respondents stating that tick bites constitute a rather high or very high health risk is 43%, which is considerably higher than respondents’ perceived health risk associated with traffic accidents (30%). This is inconsistent with objective risk measures. In 2013, road traffic accidents in Sweden caused 260 fatalities, 2 700 serious injuries and 17 500 mild injuries [51]. In comparison, there are 200–300 reported cases of TBE per year in Sweden with 1–2 fatal cases [44]. LB is much more frequent but also usually a much less serious disease, curable with antibiotics. Similar biases in risk perceptions have been found in many fields, for example in the transport sector, where travel by car is perceived as safer than by commercial airlines [17]. A framing of tick-borne diseases as a new risk and as uncontrollable, high impact-low probability events may partly explain these high risk perceptions [4, 5].

In line with earlier studies, we find significant positive associations between risk perception and the use of protective measures [21, 34, 36, 37]. However, we identify important differences between how the perceived seriousness of a single tick bite and the (lifetime) health risk from tick bites are associated with exposure to ticks and the use of protective measures. There is a significant negative association between exposure and the perceived seriousness of a tick bite, indicating that people seem to get used to having tick bites and learn that the probability of falling ill from a single tick bite is low. We find a positive association between the perceived health risk from tick bites and exposure, indicating that people also learn that the cumulative effect of repeated tick bites constitutes a serious health risk.

Our analysis of exposure and risk perceptions indicate that there are groups of respondents that can be characterized as risk deniers and risk alarmists, respectively [22]. The negative association between a perception that a tick bite is rather or very serious and belonging to the group of respondents who despite high exposure never or rarely use protective measures indicates risk denial. The high average risk perceptions found among the respondents indicate that there may be a segment of the population who could be characterised as risk alarmists.

While promoting increased awareness about risks could be an area of policy intervention, a key challenge in providing advice related to ticks, TBE and LB is how to encourage precaution without causing alarm so that engagement—which may have associated health benefits—rather than avoidance of outdoor recreational activities is promoted [52].

In line with earlier studies, we find positive and statistically significant associations between the level of knowledge about tick-borne diseases, a perception that a protective measure is effective, and the use of both protective clothing and tick checks. Consequently, one way of increasing the use of protective measures could be to actively inform people of the effectiveness of the different measures. Targeting groups with high exposure to ticks may be especially important. While only a few randomized control trials of information campaigns and education interventions exist, there are indications that information about risks and risk-reducing measures can induce an increase in the use of protective measures against tick-borne diseases [53]. However, earlier studies find important barriers to increased use of protective clothing and repellents [21, 37, 50], so expected success of such interventions should be modest. Given the high exposure to tick bites and the growing incidence of TBE and LB, other preventive measures, including vaccination programmes, should be further discussed [25, 54]. Subsidized TBE vaccination programmes have been successful in Austria and in highly endemic areas of Finland, and similar programmes may be cost effective also in other contexts [11, 46, 48, 55]. After failed attempts to introduce a vaccine against LB in the US in the early 2000s, new attempts have been made to introduce such a vaccine, which may provide effective protection for certain groups at high risk of LB [56].

There are several limitations to this study. A cross-sectional study can provide rich baseline data and identify statistically significant associations between variables but cannot determine causality. We also acknowledge the potential endogeneity between protective behaviour, risk perceptions and exposure. Our survey’s low response rate could imply a sample selection bias. Estimated parameter values should hence be considered approximations. As this is the first national survey of risk perceptions and protective behaviour related to tick-borne diseases in Sweden, there is a lack of comparative data to assess the magnitude and direction of this potential sample selection bias. However, a comparison with other studies regarding the share of TBE-vaccinated respondents gives no reason to believe that the respondents to this survey are more likely than the population in general to protect themselves against ticks.

## Supporting information

S1 Table Extended descriptive statistics and definitions of dependent variables. (DOCX)

S2 Table Extended descriptive statistics and definitions of independent variables. (DOCX)

S3 Table Logit model analysis of factors associated with five different protective measures. (DOCX)

S4 Table Count model analysis of factors associated with protective measures. (DOCX)

S5 Table Analysis of factors associated with two measures of risk perception. (DOCX)

S6 Table Multinomial logit model analysis of factors associated with protective measures. (DOCX)

S7 Table Analysis of protective behaviour using alternative coding of dummy variables for protective behavior. (DOCX)

S1 Text Survey. (PDF)
