# Supplementary material for: Learning to live with ticks? The role of exposure and risk perceptions in protective behaviour against tick-borne diseases
Source: PLoS One. 2018 Jun 20;13(6):e0198286. doi: 10.1371/journal.pone.0198286 (PMC6010238; doi:10.1371/journal.pone.0198286)
Supplement: S1 Table — (DOCX) [file pone.0198286.s001.docx]

**S1 Table Extended descriptive statistics and definitions of dependent variables**

| **Protective measures** | **Mean** | **S.d.^a^** | **Variable definitions** |
| --- | --- | --- | --- |
| *Use of specific measures ‘often’ or ‘always’* |  |  |  |
| Covering clothing | 0.644 |  | =1 if the respondent states that he or she uses the protective measure ‘often’ or ‘always’ and zero (0) if ‘never’ or ‘rarely’. |
| Check body for ticks | 0.630 |  |  |
| Tuck trousers into socks | 0.179 |  |  |
| Repellent | 0.163 |  |  |
| Avoid tall grass and bushes while in areas with ticks | 0.481 |  |  |
| *Count variables* |  |  |  |
| No. of protective measures used often/always (0–5) | 2.098 | 1.321 | =0–5 depending on the number of protective measures used ‘often’ or ‘always’ |
| No. and frequency of protective measures used (0–15) | 6.457 | 2.860 | =the number of protective measures used * the frequency. Number=0–5; Frequency=0–3 (0=never; 1=rarely; 2=often; 3=always). Min=0; Max=15 |
| *Protective measures in combination^b^* |  |  |  |
| Noprotection | 0.133 |  | No protective measure used ‘often’ or ‘always’ |
| Protclothesonly | 0.078 |  | Protective clothes is the only measure used ‘often’ or ‘always’. |
| Checkbodyonly | 0.085 |  | Tick checks is the only measure used ‘often’ or ‘always’. |
| Clothes&Body | 0.111 |  | Protective clothes in combination with tick checks used ‘often’ or ‘always’. |
| Clothes&Avoid | 0.069 |  | Protective clothes in combination with avoiding tall grass and bushes used ‘often’ or ‘always’. |
| Body&Avoid | 0.066 |  | Tick checks in combination with avoiding tall grass and bushes used ‘often’ or ‘always’. |
| Clothes&Body&Avoid | 0.142 |  | Protective clothes, tick checks and avoiding tall grass and bushes used ‘often’ or ‘always’. |
| Clothes&Body&Avoid&Other | 0.134 |  | Protective clothes, tick checks and avoiding tall grass and bushes is used ‘often’ or ‘always’ in combination with tucking trousers into socks and/or repellent. |
| Clothes&Body&Socks&Other | 0.068 |  | Protective clothes, tick checks, and tucking trousers into socks is used ‘often’ or ‘always’ in combination with repellent and/or avoiding tall grass and bushes. |
| Other measures and combinations | 0.113 |  | Specific measures or combinations used by less than 5% of the respondents. |
| Observations | 1 510 |  |  |

Notes:

^a^ Standard deviations are only displayed for variables that are not dummy variables.

^b^ These variables are used in the multinomial logit analysis (see S6 Table). They take the value of 1 if the protective measure or the combination of measures is used ‘often’ or ‘always’, and zero (0) if ‘never’ or ‘rarely’.
