# Supplementary material for: Learning to live with ticks? The role of exposure and risk perceptions in protective behaviour against tick-borne diseases
Source: PLoS One. 2018 Jun 20;13(6):e0198286. doi: 10.1371/journal.pone.0198286 (PMC6010238; doi:10.1371/journal.pone.0198286)
Supplement: S2 Table — (DOCX) [file pone.0198286.s002.docx]

**S2 Table Extended descriptive statistics and definitions of independent variables**

| VARIABLES^a^ | Mean | Obs. |
| --- | --- | --- |
| *Demographic characteristics (D)* |  |  |
| Female respondent | 0.538 | 1510 |
| Age 18–30 | 0.149 | 1510 |
| Age 31–45 | 0.257 | 1510 |
| Age 46–65 | 0.306 | 1510 |
| Age > 65 | 0.288 | 1510 |
| Household pre-tax income/month (SEK) | 44.0 | 1510 |
| Has studied at university | 0.523 | 1502 |
| Has child under 18 years | 0.262 | 1510 |
| Lives in the countryside | 0.087 | 1510 |
| *Objective risk variables (R)* |  |  |
| Lives in emerging risk area | 0.124 | 1510 |
| Lives in tick risk area | 0.585 | 1510 |
| Lives in TBE risk area | 0.291 | 1510 |
| *Exposure variables (E)* |  |  |
| Cat owner | 0.164 | 1510 |
| Dog owner | 0.175 | 1510 |
| Other outdoor animal | 0.038 | 1510 |
| Spends time in a summer home in area with TBE | 0.169 | 1489 |
| Work involves risk of tick bites | 0.099 | 1501 |
| Visits areas with ticks monthly | 0.205 | 1498 |
| Visits areas with ticks weekly | 0.366 | 1498 |
| Visits areas with ticks daily | 0.266 | 1498 |
| Visits areas with risk of TBE monthly | 0.114 | 1510 |
| Visits areas with risk of TBE weekly | 0.138 | 1510 |
| Visits areas with risk of TBE daily | 0.119 | 1510 |
| Has never had a tick bite | 0.320 | 1510 |
| Has had 1 tick bite in lifetime | 0.121 | 1510 |
| Has had 2–10 tick bite in lifetime | 0.388 | 1510 |
| Has had more than 10 tick bites in lifetime | 0.171 | 1510 |
| Had at least 1 tick bite in last 12 months | 0.311 | 1510 |
| Diagnosed with LB | 0.113 | 1510 |
| Diagnosed with TBE | 0.005 | 1510 |
| Diagnosed with other tick-borne disease | 0.002 | 1510 |
| *Knowledge variables (K)* |  |  |
| Knowledge: Low risk of getting ill from tick bite | 0.593 | 1510 |
| Knowledge: LB not contagious from person to person | 0.821 | 1510 |
| Knowledge: Mosquito repellent also repels ticks | 0.181 | 1510 |
| Knowledge: There is a vaccine against TBE | 0.613 | 1510 |
| Knowledge: TBE cannot be treated with antibiotics | 0.322 | 1510 |
| Knowledge: LB can be treated with antibiotics | 0.666 | 1510 |
| Knowledge: LB is more common than TBE in Sweden | 0.625 | 1510 |
| *Risk perception variables (P)* |  |  |
| Perception: tick bites rather or very high risk to health | 0.428 | 1510 |
| Perception: rather or very serious to get tick bite | 0.419 | 1510 |
| Perception: Checking body for ticks is very effective protection | 0.698 | 1509 |
| Perception: Protective clothing is very effective protection | 0.440 | 1510 |
| Perception: Avoiding tall grass and bushes is very effective protection | 0.470 | 1503 |
| Perception: Tucking trousers into socks is very effective protection | 0.309 | 1503 |
| Perception: Using repellent is very effective protection | 0.083 | 1503 |
| *TBE vaccination* Vaccinated against TBE | 0.245 | 1474 |

^a^ All variables are dummy variables except for household income. The standard deviation for household income is SEK 23 000; min=SEK 5 000; max=SEK 115 000. Respondents indicated their income in intervals of SEK 10 000. The mean income is generated from the mean in each interval.
