# Supplementary material for: Learning to live with ticks? The role of exposure and risk perceptions in protective behaviour against tick-borne diseases
Source: PLoS One. 2018 Jun 20;13(6):e0198286. doi: 10.1371/journal.pone.0198286 (PMC6010238; doi:10.1371/journal.pone.0198286)
Supplement: S3 Table — (DOCX) [file pone.0198286.s003.docx]

**S3 Table. Logit model analysis of factors associated with five different protective measures against tick bites and tick-borne diseases**

(marginal probabilities after logit evaluated at sample means)

|  | (1) | (2) | (3) | (4) | (5) | (6) | (7) | (8) | (9) |
| --- | --- | --- | --- | --- | --- | --- | --- | --- | --- |
| VARIABLES | Check skin | Check skin | Check skin | Prot.  Clothes | Prot.  Clothes | Prot.  Clothes | Socks | Socks | Socks |
|  |  |  |  |  |  |  |  |  |  |
| Female respondent | 0.183*** | 0.128*** | 0.131*** | 0.072*** | 0.041 | 0.048* | 0.203*** | 0.186*** | 0.191*** |
|  | (0.028) | (0.029) | (0.030) | (0.026) | (0.027) | (0.028) | (0.018) | (0.019) | (0.020) |
| Age 18–30 | -0.058 | -0.031 | -0.028 | -0.112** | -0.097** | -0.104** | -0.039 | -0.032 | -0.028 |
|  | (0.050) | (0.048) | (0.052) | (0.047) | (0.047) | (0.050) | (0.027) | (0.027) | (0.028) |
| Age 46–65 | -0.025 | -0.035 | -0.043 | -0.016 | -0.024 | -0.040 | 0.001 | -0.005 | -0.015 |
|  | (0.041) | (0.041) | (0.044) | (0.038) | (0.038) | (0.041) | (0.026) | (0.026) | (0.026) |
| Age > 65 | -0.069 | -0.092** | -0.110** | -0.017 | -0.032 | -0.060 | -0.034 | -0.043 | -0.053* |
|  | (0.044) | (0.045) | (0.048) | (0.041) | (0.041) | (0.044) | (0.027) | (0.026) | (0.028) |
| Household pre-tax income/month (SEK) | -0.001 | -0.001 | -0.001 | -0.002*** | -0.002*** | -0.002*** | -0.001** | -0.001** | -0.001* |
|  | (0.001) | (0.001) | (0.001) | (0.001) | (0.001) | (0.001) | (0.000) | (0.000) | (0.000) |
| Has child under 18 years old | 0.010 | 0.015 | 0.008 | -0.024 | -0.025 | -0.027 | -0.001 | -0.004 | -0.003 |
|  | (0.037) | (0.037) | (0.040) | (0.035) | (0.035) | (0.037) | (0.025) | (0.024) | (0.026) |
| Lives in the countryside/small village | -0.028 | -0.036 | -0.048 | -0.016 | -0.021 | -0.005 | 0.006 | 0.004 | 0.008 |
|  | (0.031) | (0.031) | (0.035) | (0.028) | (0.028) | (0.031) | (0.019) | (0.019) | (0.021) |
| Monthly or more frequent visits to areas with ticks | 0.152*** | 0.122*** | 0.108** | 0.097*** | 0.082** | 0.083** | -0.005 | -0.009 | 0.003 |
|  | (0.042) | (0.042) | (0.044) | (0.037) | (0.038) | (0.041) | (0.026) | (0.027) | (0.026) |
| Monthly or more frequent visits to areas with TBE risk | 0.114*** | 0.088*** | 0.105*** | -0.048* | -0.066** | -0.056* | 0.015 | 0.008 | 0.019 |
|  | (0.029) | (0.030) | (0.031) | (0.028) | (0.028) | (0.030) | (0.020) | (0.020) | (0.022) |
| 1 tick bite in lifetime | 0.094** | 0.082** | 0.092** | 0.035 | 0.031 | 0.018 | 0.029 | 0.026 | 0.031 |
|  | (0.038) | (0.039) | (0.040) | (0.042) | (0.042) | (0.046) | (0.033) | (0.033) | (0.035) |
| 2–10 tick bites in lifetime | 0.220*** | 0.199*** | 0.199*** | 0.007 | -0.003 | -0.013 | 0.036 | 0.035 | 0.030 |
|  | (0.030) | (0.031) | (0.033) | (0.033) | (0.034) | (0.036) | (0.024) | (0.025) | (0.026) |
| >10 tick bites in lifetime | 0.314*** | 0.290*** | 0.286*** | -0.023 | -0.040 | -0.040 | 0.064* | 0.066* | 0.064 |
|  | (0.026) | (0.028) | (0.030) | (0.041) | (0.044) | (0.047) | (0.035) | (0.037) | (0.040) |
| Lives in tick risk area | 0.218*** | 0.184*** | 0.190*** | 0.001 | -0.019 | 0.004 | -0.040 | -0.047 | -0.055* |
|  | (0.046) | (0.046) | (0.049) | (0.041) | (0.042) | (0.044) | (0.030) | (0.031) | (0.032) |
| Lives in TBE risk area | 0.197*** | 0.168*** | 0.147*** | 0.032 | 0.013 | 0.020 | 0.003 | -0.005 | -0.017 |
|  | (0.042) | (0.043) | (0.046) | (0.045) | (0.046) | (0.048) | (0.032) | (0.031) | (0.032) |
| Perception: Tick bites rather or very high risk to health |  | 0.132*** | 0.140*** |  | 0.056** | 0.049* |  | 0.016 | 0.021 |
|  |  | (0.029) | (0.030) |  | (0.028) | (0.029) |  | (0.019) | (0.020) |
| Perception: Rather or very serious to get tick bite |  | 0.102*** | 0.089*** |  | 0.078*** | 0.059** |  | 0.058*** | 0.049** |
|  |  | (0.029) | (0.031) |  | (0.027) | (0.028) |  | (0.020) | (0.020) |
| No. of correct answers on knowledge questions |  | 0.033*** | 0.031*** |  | 0.016** | 0.011 |  | 0.006 | 0.005 |
|  |  | (0.008) | (0.009) |  | (0.008) | (0.008) |  | (0.005) | (0.006) |
| Perception: Checking body for ticks is very effective protection |  |  | 0.204*** |  |  | 0.057* |  |  | 0.008 |
|  |  |  | (0.036) |  |  | (0.032) |  |  | (0.022) |
| Perception: Protective clothing is very effective protection |  |  | -0.040 |  |  | 0.129*** |  |  | 0.010 |
|  |  |  | (0.035) |  |  | (0.032) |  |  | (0.022) |
| Perception: Avoiding tall grass and bushes is very effective protection |  |  | -0.031 |  |  | -0.007 |  |  | -0.023 |
|  |  |  | (0.033) |  |  | (0.031) |  |  | (0.021) |
| Perception: Tucking trousers into socks is very effective protection |  |  | 0.045 |  |  | 0.006 |  |  | 0.081*** |
|  |  |  | (0.038) |  |  | (0.036) |  |  | (0.028) |
| Perception: Using repellent is very effective protection |  |  | 0.048 |  |  | 0.014 |  |  | -0.016 |
|  |  |  | (0.054) |  |  | (0.051) |  |  | (0.032) |
| Has studied at university |  |  | -0.036 |  |  | -0.028 |  |  | -0.044** |
|  |  |  | (0.030) |  |  | (0.027) |  |  | (0.019) |
| Has cat, dog or other outdoor animal |  |  | -0.027 |  |  | -0.053* |  |  | -0.032* |
|  |  |  | (0.034) |  |  | (0.031) |  |  | (0.019) |
| Spends time in a summer home in area with TBE |  |  | -0.015 |  |  | -0.013 |  |  | -0.001 |
|  |  |  | (0.044) |  |  | (0.037) |  |  | (0.027) |
| Work involves risk of tick bites |  |  | 0.040 |  |  | 0.107*** |  |  | 0.030 |
|  |  |  | (0.050) |  |  | (0.041) |  |  | (0.034) |
| Vaccinated against TBE |  |  | 0.006 |  |  | -0.020 |  |  | -0.026 |
|  |  |  | (0.039) |  |  | (0.035) |  |  | (0.023) |
|  |  |  |  |  |  |  |  |  |  |
| Observations | 1510 | 1510 | 1416 | 1510 | 1510 | 1416 | 1510 | 1510 | 1416 |
| Pseudo-R2 | 0,161 | 0,189 | 0,217 | 0,017 | 0,027 | 0,051 | 0,098 | 0,106 | 0,134 |

Robust standard errors in parentheses; *** p<0.01, ** p<0.05, * p<0.1

**S3 Table. Logit model analysis of factors associated with five different protective measures against tick bites and tick-borne diseases (continued)**

(marginal probabilities after logit evaluated at sample means)

|  | (10) | (11) | (12) | (13) | (14) | (15) |
| --- | --- | --- | --- | --- | --- | --- |
| VARIABLES | Repellent | Repellent | Repellent | Avoid | Avoid | Avoid |
| Female respondent | 0.112*** | 0.101*** | 0.105*** | 0.102*** | 0.058** | 0.076** |
|  | (0.019) | (0.020) | (0.019) | (0.027) | (0.028) | (0.031) |
| Age 18–30 | -0.067*** | -0.064*** | -0.063*** | -0.048 | -0.032 | -0.008 |
|  | (0.023) | (0.024) | (0.023) | (0.047) | (0.048) | (0.052) |
| Age 46–65 | -0.076*** | -0.080*** | -0.096*** | -0.043 | -0.059 | -0.049 |
|  | (0.022) | (0.022) | (0.022) | (0.039) | (0.040) | (0.043) |
| Age > 65 | -0.072*** | -0.079*** | -0.098*** | -0.032 | -0.064 | -0.076 |
|  | (0.024) | (0.024) | (0.023) | (0.042) | (0.043) | (0.047) |
| Household pre-tax income/month (SEK) | -0.000 | -0.000 | -0.000 | -0.001 | -0.001 | -0.000 |
|  | (0.000) | (0.000) | (0.000) | (0.001) | (0.001) | (0.001) |
| Has child under 18 years | -0.046** | -0.046** | -0.044* | 0.015 | 0.007 | 0.041 |
|  | (0.023) | (0.023) | (0.023) | (0.037) | (0.038) | (0.041) |
| Lives in the countryside/small village | -0.024 | -0.022 | -0.015 | -0.111*** | -0.124*** | -0.142*** |
|  | (0.020) | (0.019) | (0.021) | (0.029) | (0.029) | (0.033) |
| Monthly or more frequent visits to areas with ticks | 0.028 | 0.023 | 0.028 | -0.045 | -0.059 | -0.054 |
|  | (0.024) | (0.024) | (0.024) | (0.038) | (0.039) | (0.042) |
| Monthly or more frequent visits to areas with TBE risk | 0.026 | 0.024 | 0.025 | -0.003 | -0.028 | -0.018 |
|  | (0.021) | (0.021) | (0.021) | (0.029) | (0.030) | (0.033) |
| 1 tick bite in lifetime | -0.001 | -0.006 | -0.008 | 0.044 | 0.045 | 0.094* |
|  | (0.030) | (0.030) | (0.031) | (0.045) | (0.046) | (0.050) |
| 2–10 tick bites in lifetime | -0.001 | -0.008 | -0.005 | 0.014 | 0.020 | 0.044 |
|  | (0.024) | (0.024) | (0.024) | (0.034) | (0.036) | (0.039) |
| >10 tick bites in lifetime | 0.009 | -0.002 | 0.006 | -0.104** | -0.096** | -0.041 |
|  | (0.030) | (0.030) | (0.033) | (0.042) | (0.045) | (0.052) |
| Lives in tick risk area | -0.072** | -0.082*** | -0.085*** | 0.078* | 0.058 | 0.066 |
|  | (0.031) | (0.031) | (0.031) | (0.044) | (0.045) | (0.049) |
| Lives in TBE risk area | -0.060** | -0.067** | -0.072*** | 0.108** | 0.089* | 0.097* |
|  | (0.027) | (0.027) | (0.027) | (0.048) | (0.050) | (0.053) |
| Perception: Tick bites rather or very high risk to health |  | -0.012 | 0.000 |  | 0.054* | 0.066** |
|  |  | (0.020) | (0.020) |  | (0.030) | (0.033) |
| Perception: Rather or very serious to get tick bite |  | 0.027 | 0.017 |  | 0.168*** | 0.148*** |
|  |  | (0.020) | (0.020) |  | (0.028) | (0.030) |
| No. of correct answers on knowledge questions |  | 0.017*** | 0.014** |  | 0.011 | 0.008 |
|  |  | (0.006) | (0.006) |  | (0.008) | (0.009) |
| Perception: Checking body for ticks is very effective protection |  |  | 0.023 |  |  | -0.075** |
|  |  |  | (0.021) |  |  | (0.035) |
| Perception: Protective clothing is very effective protection |  |  | 0.047** |  |  | 0.025 |
|  |  |  | (0.024) |  |  | (0.037) |
| Perception: Avoiding tall grass and bushes is very effective protection |  |  | -0.030 |  |  | 0.251*** |
|  |  |  | (0.021) |  |  | (0.032) |
| Perception: Tucking trousers into socks is very effective protection |  |  | -0.028 |  |  | 0.041 |
|  |  |  | (0.024) |  |  | (0.040) |
| Perception: Using repellent is very effective protection |  |  | 0.240*** |  |  | 0.004 |
|  |  |  | (0.053) |  |  | (0.057) |
| Has studied at university |  |  | -0.024 |  |  | -0.023 |
|  |  |  | (0.019) |  |  | (0.030) |
| Has cat, dog or other outdoor animal |  |  | -0.050*** |  |  | -0.043 |
|  |  |  | (0.019) |  |  | (0.033) |
| Spends time in a summer home in area with TBE |  |  | -0.009 |  |  | -0.078* |
|  |  |  | (0.025) |  |  | (0.041) |
| Work involves risk of tick bites |  |  | -0.019 |  |  | 0.039 |
|  |  |  | (0.029) |  |  | (0.049) |
| Vaccinated against TBE |  |  | 0.031 |  |  | -0.003 |
|  |  |  | (0.025) |  |  | (0.038) |
| Observations | 1510 | 1510 | 1416 | 1510 | 1510 | 1416 |
| Pseudo-R2 | 0,041 | 0,049 | 0,093 | 0,026 | 0,047 | 0,098 |

Robust standard errors in parentheses; *** p<0.01, ** p<0.05, * p<0.1
