# Supplementary material for: Learning to live with ticks? The role of exposure and risk perceptions in protective behaviour against tick-borne diseases
Source: PLoS One. 2018 Jun 20;13(6):e0198286. doi: 10.1371/journal.pone.0198286 (PMC6010238; doi:10.1371/journal.pone.0198286)
Supplement: S4 Table — (DOCX) [file pone.0198286.s004.docx]

**S4 Table. Count model analysis of factors associated with protective measures against tick bites and tick-borne diseases**

|  | (1) | (2) | (3) | (4) | (5) | (6) |
| --- | --- | --- | --- | --- | --- | --- |
| VARIABLES | Protect  0–5 ^a^ | Protect  0–5 ^a^ | Protect  0–5 ^a^ | Protect  0–15 ^b^ | Protect  0–15 ^b^ | Protect  0–15 ^b^ |
| Female respondent | 0.638*** | 0.488*** | 0.508*** | 1.453*** | 1.123*** | 1.118*** |
|  | (0.065) | (0.068) | (0.069) | (0.142) | (0.146) | (0.149) |
| Age 18–30 | -0.306*** | -0.238** | -0.221** | -0.786*** | -0.642*** | -0.561** |
|  | (0.103) | (0.103) | (0.105) | (0.223) | (0.220) | (0.231) |
| Age 46–65 | -0.162* | -0.203** | -0.241*** | -0.182 | -0.279 | -0.358* |
|  | (0.091) | (0.088) | (0.091) | (0.196) | (0.190) | (0.197) |
| Age > 65 | -0.220** | -0.295*** | -0.374*** | -0.488** | -0.660*** | -0.821*** |
|  | (0.095) | (0.093) | (0.095) | (0.209) | (0.203) | (0.211) |
| Household pre-tax income/month (SEK) | -0.004*** | -0.004*** | -0.003** | -0.008** | -0.009*** | -0.007** |
|  | (0.001) | (0.001) | (0.001) | (0.003) | (0.003) | (0.003) |
| Has child under 18 years | -0.053 | -0.067 | -0.043 | -0.025 | -0.054 | 0.007 |
|  | (0.088) | (0.085) | (0.088) | (0.188) | (0.181) | (0.190) |
| Lives in the countryside/small village | -0.156** | -0.167** | -0.150** | -0.352** | -0.381** | -0.339** |
|  | (0.070) | (0.068) | (0.073) | (0.154) | (0.151) | (0.161) |
| Monthly or more frequent visits to areas with ticks | 0.212** | 0.150 | 0.155 | 0.315 | 0.180 | 0.156 |
|  | (0.101) | (0.102) | (0.102) | (0.227) | (0.229) | (0.231) |
| Monthly or more frequent visits to areas with TBE risk | 0.092 | 0.018 | 0.066 | 0.199 | 0.034 | 0.098 |
|  | (0.068) | (0.068) | (0.072) | (0.146) | (0.145) | (0.152) |
| 1 tick bite in lifetime | 0.222* | 0.184 | 0.208* | 0.598** | 0.534** | 0.535** |
|  | (0.124) | (0.121) | (0.123) | (0.263) | (0.255) | (0.256) |
| 2–10 tick bites in lifetime | 0.282*** | 0.235*** | 0.238** | 0.650*** | 0.567*** | 0.537*** |
|  | (0.090) | (0.091) | (0.093) | (0.190) | (0.190) | (0.193) |
| >10 tick bites in lifetime | 0.307*** | 0.237** | 0.292** | 0.613*** | 0.490** | 0.511** |
|  | (0.113) | (0.117) | (0.125) | (0.235) | (0.242) | (0.255) |
| Lives in tick risk area | 0.188 | 0.102 | 0.101 | 0.562** | 0.376 | 0.383 |
|  | (0.125) | (0.122) | (0.121) | (0.270) | (0.263) | (0.265) |
| Lives in TBE risk area | 0.301** | 0.208 | 0.161 | 0.893*** | 0.691** | 0.597** |
|  | (0.143) | (0.137) | (0.135) | (0.315) | (0.303) | (0.300) |
| Perception: Tick bites rather or very high risk to health |  | 0.207*** | 0.224*** |  | 0.441*** | 0.484*** |
|  |  | (0.069) | (0.069) |  | (0.148) | (0.147) |
| Perception: Rather or very serious to get tick bite |  | 0.397*** | 0.313*** |  | 0.919*** | 0.740*** |
|  |  | (0.070) | (0.069) |  | (0.150) | (0.149) |
| No. of correct answers on knowledge questions |  | 0.077*** | 0.061*** |  | 0.166*** | 0.126*** |
|  |  | (0.020) | (0.020) |  | (0.041) | (0.043) |
| Perception: Checking body for ticks is very effective protection |  |  | 0.200** |  |  | 0.560*** |
|  |  |  | (0.079) |  |  | (0.169) |
| Perception: Protective clothing is very effective protection |  |  | 0.176** |  |  | 0.152 |
|  |  |  | (0.076) |  |  | (0.167) |
| Perception: Avoiding tall grass and bushes is very effective protection |  |  | 0.139** |  |  | 0.235 |
|  |  |  | (0.069) |  |  | (0.151) |
| Perception: Tucking trousers into socks is very effective protection |  |  | 0.106 |  |  | 0.480*** |
|  |  |  | (0.080) |  |  | (0.177) |
| Perception: Using repellent is very effective protection |  |  | 0.232* |  |  | 0.744*** |
|  |  |  | (0.119) |  |  | (0.272) |
| Has studied at university |  |  | -0.142** |  |  | -0.383*** |
|  |  |  | (0.065) |  |  | (0.144) |
| Has cat, dog or other outdoor animal |  |  | -0.186*** |  |  | -0.329** |
|  |  |  | (0.071) |  |  | (0.156) |
| Spends time in a summer home in area with TBE |  |  | -0.085 |  |  | -0.123 |
|  |  |  | (0.084) |  |  | (0.181) |
| Work involves risk of tick bites |  |  | 0.175* |  |  | 0.245 |
|  |  |  | (0.103) |  |  | (0.211) |
| Vaccinated against TBE |  |  | -0.027 |  |  | 0.132 |
|  |  |  | (0.079) |  |  | (0.167) |
| Observations | 1510 | 1510 | 1416 | 1510 | 1510 | 1416 |
| Pseudo-R2 | 0.024 | 0.034 | 0.045 | 0.023 | 0.032 | 0.045 |

Robust standard errors in parentheses; *** p<0.01, ** p<0.05, * p<0.1

^a^ Count variable estimated with poisson

^b^ Count variable estimated with negative binomial regression
