# Supplementary material for: Learning to live with ticks? The role of exposure and risk perceptions in protective behaviour against tick-borne diseases
Source: PLoS One. 2018 Jun 20;13(6):e0198286. doi: 10.1371/journal.pone.0198286 (PMC6010238; doi:10.1371/journal.pone.0198286)
Supplement: S5 Table — (DOCX) [file pone.0198286.s005.docx]

**S5 Table. Analysis of factors associated with two measures of risk perception**

(marginal probabilities after logit evaluated at sample means)

|  | (1) | (2) |
| --- | --- | --- |
| VARIABLES | Serious to get one tick bite ^a^ | Health risk from tick bites ^b^ |
|  |  |  |
| Female respondent | 0.193*** | 0.203*** |
|  | (0.026) | (0.027) |
| Age 18–30 | -0.050 | -0.121*** |
|  | (0.047) | (0.045) |
| Age 46–65 | 0.093** | -0.032 |
|  | (0.041) | (0.040) |
| Age > 65 | 0.187*** | -0.015 |
|  | (0.044) | (0.044) |
| Household pre-tax income/month (SEK) | -0.001 | -0.001 |
|  | (0.001) | (0.001) |
| Has child under 18 years old | 0.071* | -0.016 |
|  | (0.039) | (0.038) |
| Lives in the countryside/small village | 0.061** | 0.051* |
|  | (0.030) | (0.030) |
| Monthly or more frequent visits to areas with ticks | -0.003 | 0.183*** |
|  | (0.038) | (0.036) |
| Monthly or more frequent visits to areas with TBE risk | 0.103*** | 0.146*** |
|  | (0.030) | (0.029) |
| 1 tick bite in lifetime | -0.055 | 0.084* |
|  | (0.043) | (0.049) |
| 2–10 tick bites in lifetime | -0.137*** | 0.167*** |
|  | (0.033) | (0.036) |
| >10 tick bites in lifetime | -0.229*** | 0.296*** |
|  | (0.034) | (0.041) |
| Lives in tick risk area | 0.070 | 0.170*** |
|  | (0.044) | (0.051) |
| Lives in TBE risk area | 0.064 | 0.191*** |
|  | (0.049) | (0.057) |
|  |  |  |
| Observations | 1 510 | 1 510 |
| Pseudo-R2 | 0.062 | 0.123 |

Robust standard errors in parentheses ; *** p<0.01, ** p<0.05, * p<0.1

^a^ The dependent variable is equal to 1 if the respondent states that it is rather or very serious to get a tick bite and 0 if ‘not serious at all’ or ‘a little serious’

^b^ The dependent variable is equal to 1 if the respondent states that tick bites constitute a rather high or very high risk to the health of the respondent or the respondent’s family, 0 if ‘rather low risk’ or ‘very low risk’
