# Supplementary material for: Learning to live with ticks? The role of exposure and risk perceptions in protective behaviour against tick-borne diseases
Source: PLoS One. 2018 Jun 20;13(6):e0198286. doi: 10.1371/journal.pone.0198286 (PMC6010238; doi:10.1371/journal.pone.0198286)
Supplement: S6 Table — (DOCX) [file pone.0198286.s006.docx]

**S6 Table. Multinomial logit model analysis of factors associated with protective measures against tick bites and tick-borne diseases**

S6 Table A: Explanatory variables: demographic and exposure

|  | (1) | (2) | (3) | (4) | (5) | (6) | (7) | (8) | (9) |
| --- | --- | --- | --- | --- | --- | --- | --- | --- | --- |
| VARIABLES | Clothes | CheckBody | Clothes &Body | Clothes &Avoid | Body &Avoid | Clothes, Body&Avoid | Clothes, Body,Avoid &Other | Clothes, Body,Socks &Other | Other |
| Female respondent | -0.065*** | -0.000 | -0.023 | -0.035*** | 0.009 | -0.029 | 0.160*** | 0.039*** | 0.034* |
|  | (0.015) | (0.013) | (0.017) | (0.011) | (0.013) | (0.021) | (0.019) | (0.012) | (0.018) |
| Age 18–30 | -0.014 | -0.010 | -0.020 | -0.025* | 0.015 | 0.016 | -0.028 | -0.034** | -0.034 |
|  | (0.025) | (0.022) | (0.031) | (0.013) | (0.025) | (0.038) | (0.028) | (0.014) | (0.025) |
| Age 46–65 | 0.018 | -0.022 | 0.026 | -0.007 | -0.018 | -0.014 | 0.008 | -0.007 | -0.056** |
|  | (0.023) | (0.018) | (0.028) | (0.014) | (0.018) | (0.030) | (0.027) | (0.016) | (0.023) |
| Age > 65 | 0.033 | -0.021 | 0.032 | -0.007 | -0.037** | -0.003 | 0.001 | -0.043*** | -0.034 |
|  | (0.026) | (0.019) | (0.031) | (0.014) | (0.018) | (0.033) | (0.030) | (0.015) | (0.025) |
| Household pre-tax income/month (SEK) | 0.000 | 0.001*** | -0.000 | -0.000 | 0.000 | -0.000 | -0.001* | -0.001** | 0.001** |
|  | (0.000) | (0.000) | (0.000) | (0.000) | (0.000) | (0.000) | (0.000) | (0.000) | (0.000) |
| Has child under 18 years | -0.018 | -0.018 | 0.016 | -0.010 | 0.001 | 0.032 | 0.004 | -0.019 | -0.030 |
|  | (0.019) | (0.017) | (0.026) | (0.013) | (0.019) | (0.031) | (0.026) | (0.015) | (0.023) |
| Lives in the countryside/small village | 0.022 | 0.001 | 0.017 | -0.030*** | -0.009 | -0.032 | -0.014 | 0.027* | -0.043** |
|  | (0.016) | (0.015) | (0.019) | (0.011) | (0.015) | (0.022) | (0.019) | (0.015) | (0.019) |
| Monthly or more frequent visits to areas with ticks | 0.013 | 0.044** | 0.046* | -0.015 | -0.001 | 0.033 | -0.015 | 0.045*** | -0.037 |
|  | (0.017) | (0.018) | (0.024) | (0.014) | (0.022) | (0.029) | (0.027) | (0.014) | (0.027) |
| Monthly or more frequent visits to areas with TBE risk | -0.025* | 0.015 | 0.012 | -0.047*** | 0.040** | 0.006 | 0.013 | 0.009 | 0.001 |
|  | (0.015) | (0.015) | (0.019) | (0.012) | (0.016) | (0.022) | (0.020) | (0.013) | (0.020) |
| 1 tick bite in lifetime | -0.020 | 0.033 | 0.027 | -0.012 | 0.004 | 0.029 | 0.022 | -0.013 | -0.023 |
|  | (0.017) | (0.038) | (0.038) | (0.013) | (0.026) | (0.040) | (0.033) | (0.022) | (0.025) |
| 2–10 tick bites in lifetime | -0.056*** | 0.060** | 0.049* | -0.031*** | 0.024 | 0.046 | 0.008 | 0.016 | -0.034 |
|  | (0.015) | (0.025) | (0.026) | (0.011) | (0.019) | (0.028) | (0.023) | (0.018) | (0.021) |
| >10 tick bites in lifetime | -0.058*** | 0.161*** | 0.062* | -0.068*** | 0.007 | -0.005 | -0.000 | 0.070** | -0.070*** |
|  | (0.013) | (0.046) | (0.035) | (0.010) | (0.023) | (0.033) | (0.028) | (0.031) | (0.021) |
| Lives in tick risk area | -0.049** | 0.050 | 0.089** | -0.018 | 0.030 | 0.041 | 0.030 | 0.003 | -0.117*** |
|  | (0.021) | (0.033) | (0.040) | (0.015) | (0.029) | (0.039) | (0.033) | (0.025) | (0.029) |
| Lives in TBE risk area | -0.047*** | 0.015 | 0.094 | -0.015 | 0.034 | 0.015 | 0.060 | 0.008 | -0.115*** |
|  | (0.018) | (0.042) | (0.060) | (0.014) | (0.039) | (0.046) | (0.043) | (0.028) | (0.021) |
| Observations | 1,510 | 1,510 | 1,510 | 1,510 | 1,510 | 1,510 | 1,510 | 1,510 | 1,510 |
| Pseudo-R2 | 0.081 | 0.081 | 0.081 | 0.081 | 0.081 | 0.081 | 0.081 | 0.081 | 0.081 |

S6 Table B: Explanatory variables: demographic, exposure, risk perceptions and knowledge

|  | (1) | (2) | (3) | (4) | (5) | (6) | (7) | (8) | (9) |
| --- | --- | --- | --- | --- | --- | --- | --- | --- | --- |
| VARIABLES | Clothes | CheckBody | Clothes &Body | Clothes &Avoid | Body &Avoid | Clothes, Body&Avoid | Clothes, Body,Avoid &Other | Clothes, Body,Socks &Other | Other |
|  |  |  |  |  |  |  |  |  |  |
| Female respondent | -0.057*** | -0.000 | -0.028 | -0.034*** | -0.001 | -0.046** | 0.141*** | 0.039*** | 0.036* |
|  | (0.016) | (0.014) | (0.018) | (0.012) | (0.014) | (0.022) | (0.020) | (0.013) | (0.019) |
| Age 18 -30 | -0.016 | -0.009 | -0.016 | -0.026* | 0.022 | 0.026 | -0.017 | -0.033** | -0.036 |
|  | (0.025) | (0.023) | (0.032) | (0.013) | (0.026) | (0.040) | (0.030) | (0.015) | (0.026) |
| Age 46 -65 | 0.021 | -0.021 | 0.032 | -0.006 | -0.021 | -0.020 | 0.003 | -0.006 | -0.059** |
|  | (0.024) | (0.019) | (0.029) | (0.014) | (0.018) | (0.031) | (0.028) | (0.017) | (0.023) |
| Age > 65 | 0.041 | -0.019 | 0.040 | -0.006 | -0.042** | -0.015 | -0.009 | -0.043*** | -0.036 |
|  | (0.028) | (0.020) | (0.033) | (0.015) | (0.018) | (0.033) | (0.029) | (0.016) | (0.026) |
| Household pre-tax income/month (SEK) | 0.000 | 0.001** | -0.000 | -0.000 | 0.000 | -0.000 | -0.001** | -0.001** | 0.001** |
|  | (0.000) | (0.000) | (0.000) | (0.000) | (0.000) | (0.000) | (0.000) | (0.000) | (0.000) |
| Has child under 18 years | -0.017 | -0.017 | 0.022 | -0.011 | -0.000 | 0.032 | 0.004 | -0.018 | -0.030 |
|  | (0.019) | (0.018) | (0.027) | (0.014) | (0.019) | (0.031) | (0.026) | (0.016) | (0.023) |
| Lives in the countryside/small village | 0.026 | 0.001 | 0.016 | -0.032*** | -0.012 | -0.034 | -0.017 | 0.028* | -0.044** |
|  | (0.017) | (0.015) | (0.020) | (0.011) | (0.015) | (0.022) | (0.019) | (0.015) | (0.019) |
| Monthly or more frequent visits to areas with ticks | 0.014 | 0.043** | 0.039 | -0.014 | -0.009 | 0.027 | -0.028 | 0.044*** | -0.032 |
|  | (0.017) | (0.019) | (0.026) | (0.014) | (0.024) | (0.031) | (0.029) | (0.015) | (0.027) |
| Monthly or more frequent visits to areas with TBE risk | -0.020 | 0.015 | 0.008 | -0.047*** | 0.033** | -0.000 | 0.003 | 0.009 | 0.003 |
|  | (0.015) | (0.015) | (0.019) | (0.013) | (0.016) | (0.023) | (0.020) | (0.014) | (0.021) |
| 1 tick bite in lifetime | -0.020 | 0.034 | 0.022 | -0.010 | 0.004 | 0.029 | 0.017 | -0.015 | -0.019 |
|  | (0.018) | (0.039) | (0.038) | (0.014) | (0.026) | (0.041) | (0.033) | (0.022) | (0.027) |
| 2–10 tick bites in lifetime | -0.058*** | 0.060** | 0.039 | -0.027** | 0.022 | 0.047 | 0.003 | 0.012 | -0.026 |
|  | (0.016) | (0.026) | (0.027) | (0.012) | (0.020) | (0.030) | (0.024) | (0.019) | (0.022) |
| >10 tick bites in lifetime | -0.058*** | 0.162*** | 0.044 | -0.066*** | 0.005 | -0.002 | -0.003 | 0.059* | -0.059** |
|  | (0.014) | (0.049) | (0.036) | (0.011) | (0.024) | (0.036) | (0.029) | (0.031) | (0.024) |
| Lives in tick risk area | -0.045** | 0.051 | 0.087** | -0.018 | 0.024 | 0.032 | 0.018 | -0.000 | -0.117*** |
|  | (0.021) | (0.035) | (0.042) | (0.015) | (0.030) | (0.041) | (0.034) | (0.026) | (0.030) |
| Lives in TBE risk area | -0.043** | 0.015 | 0.091 | -0.014 | 0.028 | 0.008 | 0.049 | 0.007 | -0.114*** |
|  | (0.018) | (0.043) | (0.061) | (0.015) | (0.038) | (0.047) | (0.043) | (0.028) | (0.022) |
| Perception: Tick bites rather or very high risk to health | -0.017 | 0.006 | 0.045** | -0.008 | 0.034** | 0.003 | 0.039** | 0.014 | -0.045** |
|  | (0.015) | (0.015) | (0.020) | (0.012) | (0.016) | (0.023) | (0.020) | (0.014) | (0.020) |
| Perception: Rather or very serious to get tick bite | -0.031** | -0.012 | -0.027 | 0.004 | 0.027* | 0.057** | 0.055*** | -0.011 | 0.025 |
|  | (0.014) | (0.015) | (0.019) | (0.011) | (0.015) | (0.023) | (0.020) | (0.013) | (0.020) |
| No. of correct answers on knowledge questions | -0.004 | 0.000 | 0.001 | -0.005 | 0.002 | 0.012* | 0.009* | 0.003 | -0.001 |
|  | (0.004) | (0.004) | (0.006) | (0.003) | (0.004) | (0.007) | (0.006) | (0.004) | (0.006) |
|  |  |  |  |  |  |  |  |  |  |
| Observations | 1,510 | 1,510 | 1,510 | 1,510 | 1,510 | 1,510 | 1,510 | 1,510 | 1,510 |
| Pseudo-R2 | 0.096 | 0.096 | 0.096 | 0.096 | 0.096 | 0.096 | 0.096 | 0.096 | 0.096 |

Robust standard errors in parentheses; p<0.01, ** p<0.05, * p<0.1
