# Supplementary material for: Learning to live with ticks? The role of exposure and risk perceptions in protective behaviour against tick-borne diseases
Source: PLoS One. 2018 Jun 20;13(6):e0198286. doi: 10.1371/journal.pone.0198286 (PMC6010238; doi:10.1371/journal.pone.0198286)
Supplement: S7 Table — (DOCX) [file pone.0198286.s007.docx]

**S7 Table. Analysis of protective behaviour using alternative coding of dummy variables for protective behaviour**

|  | (1) | (2) | (3) | (4) | (5) |
| --- | --- | --- | --- | --- | --- |
| VARIABLES | Check skin | Prot.Clothing | Socks | Repellent | Avoid |
| Female respondent | 0.097*** | 0.034* | 0.056*** | 0.036*** | 0.032** |
|  | (0.024) | (0.020) | (0.012) | (0.009) | (0.013) |
| Age 18–30 | -0.010 | -0.055** | -0.029*** | -0.006 | -0.016 |
|  | (0.041) | (0.028) | (0.008) | (0.010) | (0.019) |
| Age 46–65 | 0.008 | -0.000 | -0.004 | 0.000 | 0.010 |
|  | (0.033) | (0.028) | (0.009) | (0.010) | (0.018) |
| Age > 65 | -0.077** | 0.002 | -0.024** | -0.004 | 0.010 |
|  | (0.034) | (0.031) | (0.009) | (0.010) | (0.020) |
| Household pre-tax income/ month (SEK 1 000) | -0.000 | -0.001** | -0.000 | -0.000 | -0.001* |
|  | (0.001) | (0.000) | (0.000) | (0.000) | (0.000) |
| Has child under 18 years | 0.017 | -0.030 | -0.008 | -0.006 | 0.025 |
|  | (0.033) | (0.026) | (0.009) | (0.008) | (0.019) |
| Lives in the countryside/small village | -0.039 | -0.008 | 0.000 | 0.006 | -0.008 |
|  | (0.024) | (0.020) | (0.008) | (0.008) | (0.012) |
| Monthly or more frequent visits to areas with ticks | -0.011 | -0.003 | 0.002 | -0.004 | -0.073*** |
|  | (0.036) | (0.026) | (0.011) | (0.010) | (0.025) |
| Monthly or more frequent visits to areas with risk of TBE | 0.026 | -0.020 | -0.004 | -0.005 | -0.003 |
|  | (0.026) | (0.021) | (0.008) | (0.008) | (0.014) |
| 1 tick bite in lifetime | 0.069 | 0.076** | 0.024 | -0.013 | 0.040* |
|  | (0.046) | (0.036) | (0.018) | (0.008) | (0.024) |
| 2–10 tick bites in lifetime | 0.133*** | -0.009 | 0.008 | -0.005 | 0.000 |
|  | (0.035) | (0.024) | (0.010) | (0.008) | (0.016) |
| >10 tick bites in lifetime | 0.265*** | -0.065** | -0.001 | -0.012 | -0.006 |
|  | (0.050) | (0.027) | (0.013) | (0.009) | (0.021) |
| Lives in tick risk area | 0.087* | -0.026 | 0.001 | -0.006 | 0.010 |
|  | (0.045) | (0.031) | (0.014) | (0.012) | (0.020) |
| Lives in TBE risk area | 0.156*** | 0.007 | 0.031 | 0.006 | 0.037 |
|  | (0.058) | (0.033) | (0.020) | (0.014) | (0.029) |
| Perception: tick bites rather or very high risk to health | 0.095*** | 0.022 | 0.000 | 0.001 | 0.008 |
|  | (0.026) | (0.021) | (0.008) | (0.008) | (0.014) |
| Perception: rather or very serious to get tick bite | 0.097*** | 0.029 | 0.017* | 0.014* | 0.055*** |
|  | (0.025) | (0.020) | (0.009) | (0.008) | (0.015) |
| No. of correct answers on knowledge questions | 0.008 | -0.004 | 0.000 | 0.003 | 0.004 |
|  | (0.007) | (0.006) | (0.002) | (0.002) | (0.004) |
| Observations | 1,510 | 1,510 | 1,510 | 1,510 | 1,510 |
| Pseudo-R2 | 0.101 | 0.034 | 0.115 | 0.080 | 0.080 |

Notes: Robust standard errors in parentheses; *** p<0.01, ** p<0.05, * p<0.1
