# Supplementary material for: Learning to live with ticks? The role of exposure and risk perceptions in protective behaviour against tick-borne diseases
Source: PLoS One. 2018 Jun 20;13(6):e0198286. doi: 10.1371/journal.pone.0198286 (PMC6010238; doi:10.1371/journal.pone.0198286)
Supplement: S1 Text — (PDF) [file pone.0198286.s008.pdf]

S1 Text. Survey Final version, October 2013
[This is a freely translated version from the original survey in Swedish. It is not formatted as it was when presented to the respondents.]
Link to the formatted web survey in Swedish:
http://www.enkatfabriken.com/survey/index.php?sid=51721&lang=sv&token=n9wqfjw9ugpukz2

GÖTEBORGS UNIVERSITET

Ticks, TBE and Lyme borreliosis

A study of the risk of tick-borne diseases and how it affects our behaviour

Ticks and the tick-borne diseases Lyme borreliosis and TBE are becoming more common in Sweden. We want to know how you are affected!

Our research will increase knowledge of how different people perceive the risk of tick-borne diseases and measures to reduce risk. The research is conducted at the University of Gothenburg with funding from the Region Västra Götaland.

You are part of a random sample of individuals participating in a web panel and therefore receive this survey. To answer the survey is voluntary.

The survey takes about 15 minutes to fill in.

Even if you think that some questions are difficult to answer precisely, answer as best you can. Your responses are valuable even if they are approximate.

The survey results will be presented at various seminars and in scientific publications.

Your answers will be treated so that unauthorized access to them will be prevented. Responsible for your personal information - in accordance with the Data Protection Act (1998: 204) – is University of Gothenburg, Privacy Officer Kristina Ullgren, Box 100, SE-405 30 Gothenburg, tel. 031-7861092, email: kristina.ullgren@gu.se.

Many thanks for your participation in our research! For additional information about the survey and its results, or if there are any questions, please feel free to contact us.

Responsible for the study:

Daniel Slunge
PhD candidate
Institutionen för nationalekonomi
Göteborgs universitet
Tel. 031-7869205
daniel.slunge@gu.se

Thomas Sterner
Profesor
Institutionen för nationalekonomi
Göteborgs universitet
Tel. 031-7861377
thomas.sterner@gu.se

Introductory questions

1. **When were you born? [scroll bar]**
2. **Sex**
   - ☐ **female**
   - ☐ **male**
3. **Area of living? [Name: of municipality in [scroll bar] (compulsory question)**
4. **Zip code: ___________**

5. **How do you rate your general health status?**

Very bad	Rather bad	Neither good or bad	Rather good	Very good
1	2	3	4	5
○	○	○	○	○

6. **How large risk do you think that the following things may have for your or your family´s health? (please tick one option per line)**

	Very low risk	Rather low risk	Rather high risk	Very high risk	No opinion
Trafik accidents					
Air pollution					
Additives or pesticide residues in food					
A new pandemic					
Side effects from vaccinations					
Tick bites					

7. **How much trust do you have in………?**

	1.Very low trust	2. Rather low trust	3. Neither low or high trust	4. Rather high trust	5.Very high trust	No opinion
The Swedish health care						
The County Council where you live						
The medical center where you are listed						

Vaccine recommendations from the health care system						

Exposure to ticks and tick-borne diseases

8. **Have you ever had a tick bite?**

☐ Yes
☐ No → Q 15
☐ Don´t know

9. [If yes] How many?

☐ 1
☐ 2-10
☐ 11-49
☐ 50 or more

10. **[If yes on Q 8] How many tick bites have you had in the last 12 months?**

☐ 0
☐ 1-2
☐ 3-5
☐ 6-10
☐ More than 10

11. In which municipality did you get bitten by ticks during the last 12 months?(several answers possible)

☐ In my home municipality
☐ In another municipality (scroll bar)
☐ In another country
☐ Don't know

12. Have you ever had a tick-borne disease?

☐ Yes
☐ No → Q 15
☐ Don´t know

13. [If yes] Which disease? (several answers possible)

☐ Lyme borrelosis
☐ TBE (tick-borne encephalitis)
☐ Other disease (specify):________________
☐ Don´t know

14. Was the disease diagnosed by a doctor?

☐ Yes

☐ No
☐ Don´t know

15. Do you have children below the age of 18 years?

☐ Yes
☐ No → Q 22

16. [If yes] Has your child/any of your children ever been bitten by a tick?

☐ Yes
☐ No
☐ Don´t know

17. **[If yes / do not know at Q16] Has your child / any of your children ever had a tick-borne disease?**

☐ Yes
☐ No → Q 22
☐ Don´t know

18. [If yes] Which disease? (several answers possible)

☐ Lyme borreliosis
☐ TBE (tick-borne encephalitis)
☐ Other disease (specify):__________________
☐ Don´t know

19. Was the disease diagnosed by a doctor?

☐ Yes
☐ No
☐ Don´t know

20. Do you have another family member or someone in your immediate circle of acquaintances who has ever had a tick-borne disease?

☐ Yes
☐ No → Q 22
☐ Don´t know

21. [If yes] Which disease? (several answers possible)

☐ Lyme borreliosis
☐ TBE (tick-borne encephalitis)
☐ Other disease (specify):__________________
☐ Don´t know

22. Do you have animals?

☐ No → Q 27
☐ Only indoor pets (including indoor cat)
☐ cat
☐ dog
☐ Other animal (e.g. horse, cow or sheep)

23. **By how many ticks has your animal been bitten during the last 12 months? (If you have more than one animal choose the one that has been bitten by most ticks)**
   - ☐ 0
   - ☐ 1
   - ☐ 2-10
   - ☐ 11-50
   - ☐ More than 50
   - ☐ Don't know

24. **How often do you check your animal for ticks during the summer?**

Daily	
1-3 times a week	
1-2 times a month	
More rarely	
Never	

25. **How often do you protect your cat or dog through using a special necklace, spot-on, a spray or other item?**
   - ☐ Always
   - ☐ Often
   - ☐ Rarely
   - ☐ Never

26. **How much did you approximately pay for this type of protection for your cat or dog during the last 12 months? (if you have several pets specify the total amount)**
   - ☐ 0 kr
   - ☐ 1-100 kr
   - ☐ 101-300 kr
   - ☐ 301-500 kr
   - ☐ 501-1000 kr
   - ☐ More than 1000 kr

Risk perception

27. **How serious do you think it is to:**

Choose the correct answer for each item:

	Not serious at all	A little serious	Rather serious	Very serious	Don´t know
Get bitten by a tick					
Get the tick-borne Lyme borrelosis					
Get TBE					

28. How likely do you think that any of the following events occur during the next 12 months?

Choose the correct answer for each item:

	Not likely at all	A little bit likely	Rather likely	Very likely	Don´t know
You get bitten by a tick					
You become ill as a result of a tick bite					
You are diagnosed with Lyme borrelosis					
You are diagnosed with TBE (tick-borne encephalitis)					

29. [IF HAVE CHILDREN] How likely do you think that any of the following events occur during the next 12 months?

	Not likely at all	A little bit likely	Rather likely	Very likely	Don´t know
Your child/ any of your children get diagnosed with Lyme Borrelosis or TBE					

30. Which of the following statements do you think are correct? (tick true or false for each statement)

	True	False	Don´t know
If you get bitten by a tick there is a large risk that you get ill	[ ]	[ ]	[ ]
Borrelia can be passed from one person to another	[ ]	[ ]	[ ]
Mosquito repellent decrease the risk of getting a tick-bite	[ ]	[ ]	[ ]
There is a vaccine you can buy that can prevent TBE	[ ]	[ ]	[ ]
TBE can be treated with antibiotics	[ ]	[ ]	[ ]
Borrelia can be treated with antibiotics	[ ]	[ ]	[ ]
Borrelia is more common than TBE in Sweden	[ ]	[ ]	[ ]

31. Information about TBE and Lyme borreliosis

Please read the following information before you proceed.

Information about TBE and Lyme borreliosis

The disease TBE

TBE (tick-borne encephalitis) is a disease that can be transmitted between animals and humans by ticks. Common symptoms of TBE are high fever, severe headache and occasional convulsions and paralysis. About 40 percent of those infected develop long-term or permanent symptoms such as fatigue, memory lapses, and in rare cases, paralysis. Today there is no cure for TBE, however, there is an effective vaccine.

Where can you get infected with TBE and how many are infected?

TBE virus are mainly found among ticks in the archipelagos of Uppland and Södermanland and in parts of Lake Mälaren. Most people get infected around Södertörn, Södertäljeviken and central parts of the Lake Mälaren. The virus is also found around Vänern and Vättern and in some places along the west coast. In 2011 and 2012, there were about 280 reported cases of TBE per year in Sweden. Each dot on the map below indicates where someone was infected with TBE in 2012.

Vaccine

It is possible to get vaccinated against TBE. Three doses of the vaccine gives an effective protection for three years among almost 100% of those who take the vaccine. The side effects from the vaccine are mild. Vaccination is usually recommended for permanent and summer residents in risk areas and to people who spend a lot of time in forest areas in TBE risk areas, and that often gets bitten by a tick.

Lyme borreliosis

Among approximately half of those infected with Lyme borreliosis a ring-shaped redness at the site of the tick bite develops. If the infection is not treated with antibiotics, it can months progress to a more severe illness with central nervous system symptoms after a couple of weeks to. You can also get joint problems and in rare cases also affect the heart. The disease can be treated with antibiotics.

Where can you get Lyme borreliosis and how many are infected?

Borrelia bacteria exists where ticks are present. There are no reliable statistics on the number of cases of Lyme borreliosis in Sweden. Studies indicate that there may be around 10 000 cases per year in southern Sweden. North of the Dalälven, there are few cases. Studies also show that even if you get bitten by a tick carrying the Borrelia bacteria, the risk of getting infected is low.

Vaccine

Today there is no vaccine for Lyme borreliosis to buy, but there are ongoing efforts to develop such a vaccine**.**

TBE cases 2012

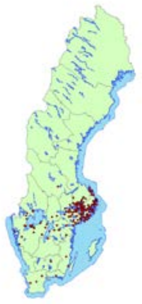

Outdoor activities

32. Is there a risk that you get bitten by a tick while working on your job?

- ☐ Yes
- ☐ No
- ☐ Don’t know

33. How many hours per day do you usually spend outdoors during the months May to September?

	0	1-3	4-8	9 or more
Weekdays				
Weekends/vacation				

34. How often did you spend time in forests or other areas where you may come into contact with ticks during the months of May to September this year?

Just select one of the following:

- ☐ Daily
- ☐ 1-3 Times a Week
- ☐ 1-2 Times a Month
- ☐ More Rarely
- ☐ Never
- ☐ Don´t know

✓ Ticks thrive in the forests and meadows with tall grass and scrubland.

35. How often, during the months of May to September this year, did you spend time in areas where you may come into contact with ticks and where you also know or have heard that there is TBE?

Just select one of the following:

- ☐ Daily
- ☐ 1-3 Times a Week
- ☐ 1-2 Times a Month
- ☐ More Rarely
- ☐ Never
- ☐ Don´t know

Each dot on the map to the right indicates where someone was infected with TBE in 2011.

Reported cases of TBE 2012

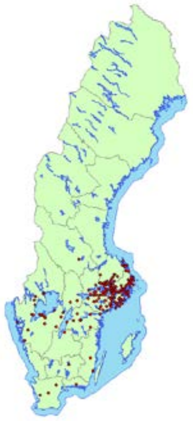

Protective measures against tick-bites

36. How often do you protect yourself against tick bites, in any of the following ways, when you are in forests or in other areas where you may get in contact with ticks? Put a cross on each line.

	Never	Rarely	Often	Always
Uses covering trousers and long-sleeved shirt / jacket	☐	☐	☐	☐
Uses anti-mosquito or tick repellant	☐	☐	☐	☐
Have socks outside the trousers	☐	☐	☐	☐
Avoid tall grass and go near the bushes	☐	☐	☐	☐
Examining body for ticks after being outdoors	☐	☐	☐	☐

37. [If child <18 years] How often do you protect yourself against tick bites, one of the following ways, when you are in areas with ticks?

	Never	Rarely	Often	Always
Examines my child's body for ticks after being outdoors	☐	☐	☐	☐

38. How good protection against tick bites do you think that the following measures provide:

	**No protection**	**Rather poor**	**Rather good**	**Very good**
Use covering trousers and long-sleeved shirt / jacket	☐	☐	☐	☐
Use anti-mosquito or tick repellant	☐	☐	☐	☐
Have socks outside the trousers	☐	☐	☐	☐
Avoid tall grass and go near the bushes	☐	☐	☐	☐
Examining body for ticks after being outdoors	☐	☐	☐	☐
[respondents with outdoor pets:]				
Examine pets for ticks when they have been outdoor	☐	☐	☐	☐

39. **Do you avoid activities or areas where there is a risk that you get bitten by ticks?** (For example, if you refrain from going for a walk in a certain forest area or if you are walking on a road instead of a path with high grass to avoid ticks)

☐ Yes, very often
☐ Yes, rather often
☐ Yes, but rarely
☐ No, never
☐ Don't know

40. **Briefly describe what type of activities or areas that you avoid:**

____

Choice between recreational areas

Imagine that it is late summer and that you have decided to spend four hours during the weekend outdoors engaging in activities such as walking, picking berries or mushrooms, picnicking, or other things you enjoy doing. Imagine that you are to choose between spending the four hours outdoors in one of two areas (area A or B).

We will now describe areas A and B and then ask you to **select the area in which you would prefer to spend the four hours**:

The following things distinguish areas A and B from each other:

Area characteristics
Imagine that you have rated the area after a previous visit. This can include how beautiful the area is, its natural values or the presence of mushrooms and berries. You have divided the areas into the following categories:
- ordinary area,
- nice area,
- very nice area.

The presence of ticks:

- **no ticks**
- **some ticks** – it is likely that you will get 1–2 ticks on your clothes or your skin if you walk in tall grass or in the forest during your stay in the area
- **many ticks** – it is likely that you will get 4 or more ticks on your clothes or your skin if you walk in tall grass or in the forest during your stay in the area

The distance to the area

- 1 km
- 5 km
- 30 km
- 70 km

The risk of contracting Lyme borreliosis and TBE
Both Areas A and B are about 10 square kilometres and are visited by about 10 000 people per year. The figure below contains 10 000 squares. Each square symbolises 1 person who visits the area.

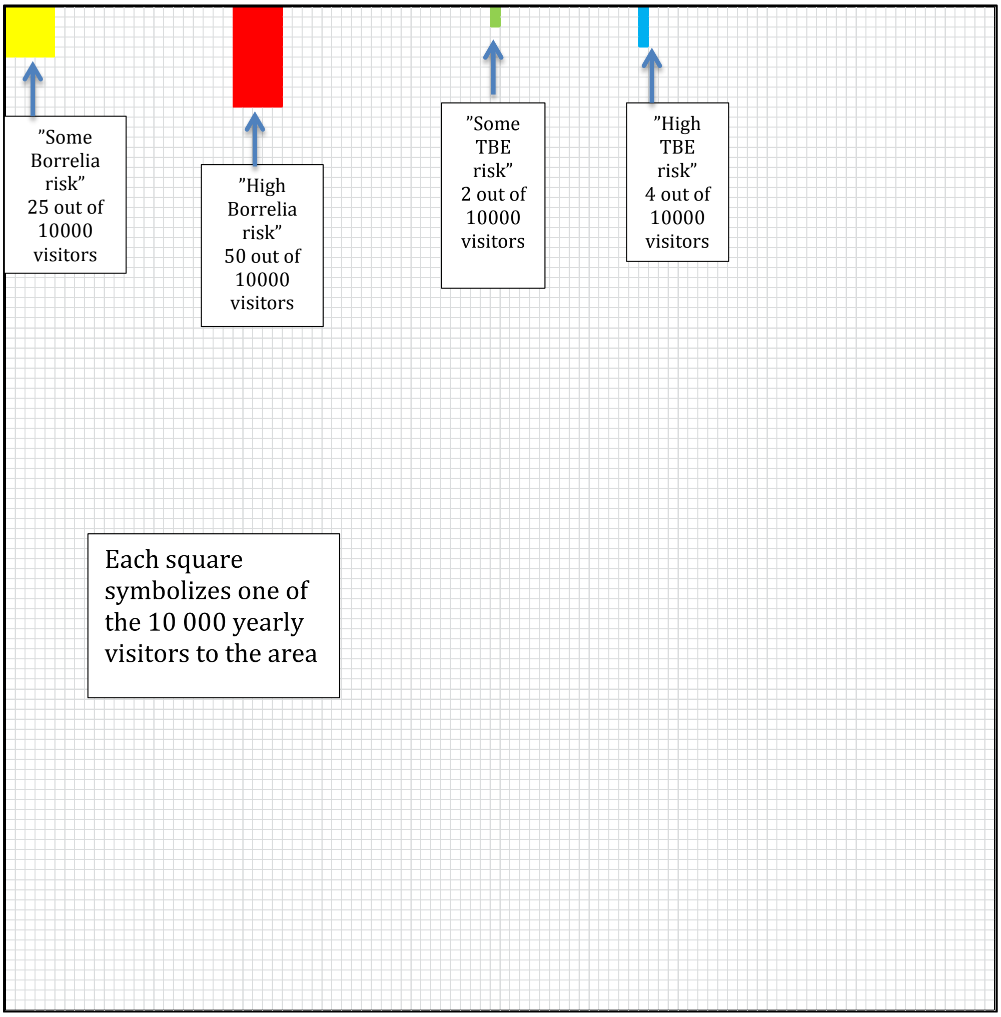

- **Some Borrelia risk** – means that 25 of the 10 000 visitors get Lyme borreliosis every year after visiting the area. This is symbolised by the 25 yellow-coloured squares in the figure.
- **High Borrelia risk** – means that 50 people get Lyme borreliosis every year after visiting the area. This is symbolised by 50 red-coloured squares in the figure.
- **Some TBE risk** – means that 2 people get TBE every year after visiting the area. This is symbolised by the 2 green-coloured squares in the figure.
- **High TBE risk**– means that 4 people get TBE every year after visiting the area. This is symbolised by the 4 blue-coloured squares in the figure.

This means that *only a very small share of the visitors become infected with Lyme borreliosis or TBE every year.*

41. To be sure that you understand the information, we ask you to answer the following question:
If we state that an area has some Borrelia risk and high TBE risk, is there then a greater risk of contracting Borrelia than TBE while visiting the area? [compulsory question]
Select just one of the following:

☐ Yes

☐ No

[If yes, the following text is shown:) Correct answer!

[If no, the following text is shown:]
- Some Borrelia risk – means that 25 of the 10 000 visitors contract Lyme borreliosis each year in conjunction with visits to the area.
- High TBE risk – means that 4 of the 10 000 visitors get infected with TBE each year in conjunction with visits to the area.
Thus, there is a greater risk of contracting Lyme borreiosis than TBE while visiting the area.

Read about what characterises areas A and B in the table below. Select whether you would choose to go to area A or B to spend the four hours outdoors. If, under the given circumstances, you would choose not to visit either of the areas, mark alternative C, 'Not go'.

Read the text in the box before making your choice:

Experiences from other similar surveys show that it is common that people make other choices in a survey than they would in real life. Some may state that they would travel 70 km to visit an area while in real life they would only be willing to travel 30 km. We want you to state the choice you would make if this was a real situation.

	**Area A**	**Area B**	**Not go (C)**
**Area characteristics**	Nice area	Nice area	
**Presence of ticks**	No ticks	Some ticks	
**Risk of Borrelia**	No Borrelia risk	Some Borrelia risk	
**Risk of TBE**	No TBE risk	Some TBE risk	
**Distance**	5 km	1 km	
**Mark if you would choose A, B or C:**	[ ]	[ ]	[ ]

We will now modify the properties of Area A and Area B. After examining the characteristics of each area we want you to select Area A or B, or the alternative C (not go). Make every choice without thinking of your previous choices.

	**Area A**	**Area B**	**Not go (C)**
**Area characteristics**	Nice area	Nice area	
**Presence of ticks**	No ticks	Many ticks	
**Risk of borrelia**	No borrelia risk	High borrelia risk	
**Risk of TBE**	No TBE risk	HighTBE risk	
**Cost**	5 km	1 km	
**Mark if you would you choose A, B or C:**	[ ]	[ ]	[ ]

Continue to make your choice after reading what applies to each area.
Make every choice without thinking of your previous choices.

	Area A	Area B	Not go (C)
**Area characteristics**	Nice area	Nice area	
**Presence of ticks**	Many ticks	Some ticks	
**Risk of borrelia**	High borrelia risk	Some borrelia risk	
**Risk of TBE**	High TBE risk	SomeTBE risk	
**Distance**	1 km	1 km	
**Mark if you would you choose A, B or C:**	[ ]	[ ]	[ ]

[The three tables above are presented to all respondents and constitute a transitivity test. All respondents are presented to four additional tables which are created using statistical design methodology and divided into four different blocks. The order in which the tables are presented are randomized among the respondents.]

Vaccine against Lyme borreliosis

Today there is no vaccine against Lyme borreliosis to buy, but there are ongoing efforts to develop such a vaccine. Imagine that there was a vaccine that gave almost 100% of those vaccinated a good protection against Lyme borreliosis and that the side effects from the vaccine were mild. As with today's TBE vaccination three doses of the vaccine would be necessary to protect ONE PERSON during at least 3 years.

42. **Would you vaccinate yourself or someone in your household against Lyme borrelosis if it cost a total of [100; 250; 500; 1000; 5000] SEK for the three doses of the vaccine that protects ONE person for at least three years' time?** *(One of the numbers in [ ] is presented to the respondent)*

PLEASE READ THE INFORMATION IN THE TEXT BOX BEFORE RESPONDING TO THE QUESTION

Experiences from other similar surveys show that it is common to make other choices in a survey than one would make in real life. Some may state that they would be willing to pay a higher price for a vaccine than they would actually pay in real life. This may be due to the fact that one does not really consider how big an impact an extra cost actually has to the family budget. We want you to state the choice you would make if this was a real situation.
Disregard any answers you gave to similar questions earlier in the survey.

☐ Yes
☐ No [jump to Q.48]

43. **[IF YES] Enter the number of adults in your household that you would choose to vaccinate at this cost: ____________________**

44. **Enter the number of children below age 18 in your household that you would choose to vaccinate at this cost: ___________________________________**

45. **The total cost to vaccinate [x] people in your household would be [yy] kr.**

You now have the possibility to adjust your choice!

46. **How certain are you that this is the choice you would make in a real world situation?**
    - ☐ Very uncertain
    - ☐ Rather uncertain
    - ☐ Rather certain
    - ☐ Very certain

47. **Do you think other persons resembling yourself (e.g. persons that have the same age, sex and lives in the same municipality as you) would choose to get vaccinated against Lyme borreliosis if it cost a total of [***SAME PRICE AS ABOVE PRESENTED***] SEK for the three doses of the vaccine that protects ONE person for at least three years' time?**
    - ☐ Yes
    - ☐ No

48. **[IF NO] Would you vaccinate yourself or someone in your household against Lyme Borreliosis if the vaccine was free of charge?**
    - ☐ Yes
    - ☐ No
    - ☐ Don´t know

49. [FOLLOW-UP QUESTION TO RESPONDENTS WHO SAYS YES TO INTEREST OF BUYING BORRELIA VACCINE] **Why would you buy a vaccine against Lyme borrelosis?** Select all that apply:
    - ☐ I often stay in areas with ticks
    - ☐ I often get bitten by ticks
    - ☐ I would not need to worry about Lyme borreliosis
    - ☐ Someone close to me (eg, friend, family member) has had Lyme borreliosis
    - ☐ I've heard a lot about Lyme borreliosis in the media
    - ☐ Other reason, please specify: ___________________________________
    - ☐ Do not know

50. [FOLLOW-UP QUESTION TO RESPONDENTS WHO SAYS NO TO INTEREST OF BUYING BORRELIA VACCINE] **Why would not you buy a vaccine against Lyme borreliosis?**

Select all that apply:
- ☐ I'm never / rarely in tick-affected areas
- ☐ The risk of contracting Lyme borreliosis is so small that I do not need the vaccine
- ☐ The vaccine costs too much
- ☐ If I become infected with Lyme borreliosis, I expect that I can treat it with antibiotics

- ☐ I am afraid of negative side effects of the vaccine
- ☐ I do not think that the vaccine would be effective
- ☐ Medical reasons
- ☐ I'm afraid of needles
- ☐ Other reason, please specify: ________________________________
- ☐ Do not know

Vaccine against TBE

51. Have you been vaccinated against TBE?
Just select one of the following:
- ☐ Yes
- ☐ Yes, I have started vaccination against TBE but not had time to take all doses
- ☐ I have started but discontinued vaccination against TBE [PROCEED TO QUESTION 52]
- ☐ No [PROCEED TO QUESTION 57]
- ☐ Don´t know

52. [IF "YES, I have started", "YES" or "I have started but discontinued vaccination against TBE"] **What year did you take your last dose of the vaccine?**
Year: (scroll bar)

53. [IF YES, I have started or YES] **Enter the number of adults in your household who are vaccinated against TBE.** Number of adults: ____________

54. [IF YES, I have started or YES] **Enter the number of children in your household who are vaccinated against TBE.**
Number of children under 18 years: ________________

55. [IF YES, I have started or YES] **What were the main reasons that you got vaccinated against TBE?**
Select all that apply:
- ☐ I live in a TBE risk area
- ☐ I travel to TBE risk areas
- ☐ I often stay in areas with ticks
- ☐ I often get bitten by ticks
- ☐ I do not need to worry anymore about TBE
- ☐ Someone close to me (eg, friend or family member) has contracted TBE
- ☐ Seen, read or heard about TBE in the media
- ☐ My doctor recommended TBE vaccination
- ☐ My family/friends recommended TBE vaccination
- ☐ Other reason, please specify: ________________________________
- ☐ Do not know

56. **Have you changed your behavior in any of the following ways after you got vaccinated against TBE?** (state to what extent you agree with the following statements, please tick one option per line)

	Don´t agree at all	Agree to some extent	Agree completely	Don´t know
I worry less about tick bites				
I spend more time in forests				
I less often do not avoid to go to areas with TBE risk				
I do not check my body for ticks after being outdoors as carefully as before				
I wear protective clothing less often when I am in forest areas				

57. [IF NO TO Q51] **Why are you not vaccinated against TBE?**

Select all that apply

- ☐ I'm never / rarely in areas with ticks
- ☐ I'm never / rarely in areas where there is a risk of contracting TBE
- ☐ The risk of contracting TBE is so small that I do not need to be vaccinated
- ☐ I did not know there was a vaccine against TBE
- ☐ The vaccine costs too much
- ☐ I have intended to get vaccinated, but have not gotten to it
- ☐ Have never thought about it
- ☐ It is complicated / take too long to get vaccinated
- ☐ I'm afraid of getting side effects from the vaccine
- ☐ I'm afraid of needles
- ☐ Medical reasons
- ☐ Other reason, please specify: ________________________________
- ☐ Do not know

58. [IF NO to Q51] **Would you vaccinate yourself or someone in your household against TBE if it cost a total of [100, 250, 500, 750, 1000] SEK for the three doses of the vaccine that protects ONE person for at least three years' time?** *(One of the numbers in [ ] is presented to the respondent)*

PLEASE READ THE INFORMATION IN THE TEXT BOX BEFORE RESPONDING TO THE QUESTION

> *Experiences from other similar surveys show that it is common to make other choices in a survey than one would make in real life. Some may state that they would be willing to pay a higher price for a vaccine than they would actually pay in real life. This may be due to the fact that one does not really consider how big an impact an extra cost actually has to the family budget. We want you to state the choice you would make if this was a real situation.*
> *Disregard any answers you gave to similar questions earlier in the survey.*

☐ Yes
☐ No [JUMP TO Q63]

59. **[IF YES] Enter the number of adults in your household that you would choose to vaccinate at this cost: ____________________**

60. **[IF YES] Enter the number of children below age 18 in your household that you would choose to vaccinate at this cost:**

    ____

The total cost to vaccinate [x] people in your household would be [yy] kr.
You now have the possibility to adjust your choice!

61. **How certain are you that this is the choice you would make in a real world situation?**
    ☐ Very uncertain
    ☐ Rather uncertain
    ☐ Rather certain
    ☐ Very certain

62. **Do you think other persons resembling yourself (e.g. persons that have the same age, sex and lives in the same municipality as you) would choose to vaccinate against TBE if it cost a total of** *[SAME PRICE AS ABOVE]* **SEK for the three doses of the vaccine that protects ONE person for at least three years' time against TBE?**
    ☐ Yes
    ☐ No

63. **[IF NO] Would you vaccinate yourself or someone in your household against TBE if the vaccine was free?**

☐ Yes
☐ No
☐ Don´t know

64. [IF "I have started but discontinued vaccination against TBE" IN Q51]
Why have not you finished your TBE vaccination?
Select all that apply

- ☐ Forgot to do it
- ☐ I have intended to continue to take TBE vaccine, but have not gotten to it
- ☐ Too busy to do a follow-up meeting
- ☐ Lack of information, not sure when I would take follow-up injections
- ☐ No longer live in a TBE-risk area
- ☐ Do not travel to TBE risk areas
- ☐ The vaccine costs to much
- ☐ I discussed it with friends / family and came to the conclusion that I do not need TBE vaccination
- ☐ I'm afraid of side effects from the vaccine
- ☐ I'm already protected with the doses I have taken, I do not take any more doses of vaccine
- ☐ Other reason, please specify: ______________________________
- ☐ Do not know

Public programs to reduce the risk of tick-borne diseases

65. Swedish authorities can take various measures to reduce the risk of tick-borne diseases. Below are a number of possible actions. What is your opinion on each of them?
Choose one answer for each item:

Program	*Very good proposal*	*Rather good proposal*	*Neither good nor bad proposal*	*Rather bad proposal*	*Very bad proposal*
Reduce the price of TBE vaccinations to people living in areas at risk					
Reduce the price of TBE vaccination to anyone who wants to get vaccinated in Sweden					
Include TBE vaccination in the general vaccination program for children					
Increase resources for research on tick-borne diseases					
Increase communication efforts on tick-borne diseases					
Drastically reducing the number of deer in Sweden by hunting (deer are an important host animals for ticks)					

Information about you and your household

66. Where do you live today? (Tick the option that best describes your living area)

- ☐ Countryside with just a few houses in sight
- ☐ Village or small town in the countryside
- ☐ Small town (less than about 50 000 inhabitants)
- ☐ Medium-sized town (about 50 000 - 200 000 residents)
- ☐ Larger city (more than 200 000 inhabitants)

67. Which people live in your household?

Select all that apply:

- ☐ I live alone
- ☐ I live with / share regularly household with adults 18 years and older
- ☐ I live with / regularly share of household with children below 18 years

68. Including yourself, how many adults aged 18 or older, are there in the household?: ____

69. How many children below 18 years are there in the household?:_________

70. Please indicate <u>YOUR</u> total monthly income (before tax).
Estimate your total income from all sources or income, such as wages, pensions, social security, unemployment compensation, net income from business, child support or any other income. (please circle one alternative)

- ☐ Less than 10 000 kronor
- ☐ 10 000 - 19 999 kronor
- ☐ 20 000 - 29 999 kronor
- ☐ 30 000 - 39 999 kronor
- ☐ 40 000 - 49 999 kronor
- ☐ 50 000 - 59 999 kronor
- ☐ 60 000 - 69 999 kronor
- ☐ 70 000 - 79 999 kronor
- ☐ Above 80 000 kronor

71. Please indicate the total monthly income of your <u>HOUSEHOLD</u> (before tax). Estimate your total household income from all sources or income, such as wages, pensions, social security, unemployment compensation, net income from business, child support or any other income. (please circle one alternative)

- ☐ Less than 10 000 kronor
- ☐ 10 000 - 19 999 kronor
- ☐ 20 000 - 29 999 kronor
- ☐ 30 000 - 39 999 kronor
- ☐ 40 000 - 49 999 kronor
- ☐ 50 000 - 59 999 kronor
- ☐ 60 000 - 69 999 kronor
- ☐ 70 000 - 79 999 kronor
- ☐ 80 000 – 89 999 kronor
- ☐ 90 000 – 99 999 kronor
- ☐ 100 000 – 110 000 kronor
- ☐ Above 110 000 kronor

72. Were you ....:

☐ Born in Sweden

☐ Born in another country

73. Do you own or have regular access to a summer house?

☐ Yes

☐ No, [GO TO QUESTION 79]

74. Where is your summer house located?

☐ In Sweden

☐ Abroad [go to Q77]

75. In which municipality? [scroll bar]

76. Which zip code? ___________________

77. In which country? (scroll bar)

78. How many days or parts of days did you spend in the summerhouse during the period May to September this year?

☐ 0 days

☐ 1-7 days

☐ 8-21 days

☐ More than 21 days

☐ Do not know

79. Do you or your household own a car?

☐ Yes, one car

☐ No, but I have regular access to a car

☐ No

80. What is the highest level of schooling you have completed?

Please circle one alternative.

☐ Not completed elementary school

☐ Elementary school

☐ Secondary school 1-2 years

☐ Secondary school 3 years

☐ University 1-3 years

☐ University more than 3 years

☐ Doctoral studies

81. Life Situation / Employment

Just select one of the following:

- ☐ Employment in the private or public sector (including sick leave, maternity leave)
- ☐ Working in own business (self employed)
- ☐ Have work in labor market programs / Undergoing employment training
- ☐ Unemployed
- ☐ Retired, Age Pensioners
- ☐ Have "sick or activity payment" (ex early retirement / disability pensioner)
- ☐ Student
- ☐ Other: ______________________________

Final questions

82. ANY OTHER COMMENTS about ticks and tick-borne diseases or about the survey?

____

____

THANK YOU FOR YOUR COOPERATION!

The questionnaire contains brief information about Lyme borreliosis and TBE. Detailed information on tick-borne diseases can be found at the following websites: www.SMI.se and www.Internetmedicin.se.

The information on Lyme borreliosis risk and TBE risk given for the hypothetical areas A and B are based on information about the areas in Sweden, with the highest incidence of Lyme borreliosis and TBE. In most places, the risk is much lower.

The actual risk of becoming infected with TBE or Lyme borreliosis is largely dependent on factors such as:

- where you live and where you are in the summer, since ticks carrying TBE or Lyme borreliosis is more common in certain parts of Sweden
- Leisure activities / work habits (if you spend much time in the forest, scrubland or high grass, the risk of becoming infected is higher)
- if you dress in protective clothing, such as pants and long sleeves , when you walk in the woods or in tall grass, the risk of becoming infected is lower
- if you check your body for ticks after being out in the woods or tall grass, the risk of becoming infected is lower
- if you are vaccinated against TBE the risk of getting TBE is minimal.
